# Current and Future Prospects for Gene Therapy for Rare Genetic Diseases Affecting the Brain and Spinal Cord

**DOI:** 10.3389/fnmol.2021.695937

**Published:** 2021-10-06

**Authors:** Thomas Leth Jensen, Casper René Gøtzsche, David P. D. Woldbye

**Affiliations:** ^1^Department of Neurology, Rigshospitalet University Hospital, Copenhagen, Denmark; ^2^Department of Neuroscience, University of Copenhagen,Copenhagen, Denmark

**Keywords:** rare diseases, gene therapy, viral vectors, spinal muscular atrophy, personalized medicine, spinal cord, central nervous system, clinical trials

## Abstract

In recent years, gene therapy has been raising hopes toward viable treatment strategies for rare genetic diseases for which there has been almost exclusively supportive treatment. We here review this progress at the pre-clinical and clinical trial levels as well as market approvals within diseases that specifically affect the brain and spinal cord, including degenerative, developmental, lysosomal storage, and metabolic disorders. The field reached an unprecedented milestone when Zolgensma® (onasemnogene abeparvovec) was approved by the FDA and EMA for *in vivo* adeno-associated virus-mediated gene replacement therapy for spinal muscular atrophy. Shortly after EMA approved Libmeldy®, an *ex vivo* gene therapy with lentivirus vector-transduced autologous CD34-positive stem cells, for treatment of metachromatic leukodystrophy. These successes could be the first of many more new gene therapies in development that mostly target loss-of-function mutation diseases with gene replacement (e.g., Batten disease, mucopolysaccharidoses, gangliosidoses) or, less frequently, gain-of-toxic-function mutation diseases by gene therapeutic silencing of pathologic genes (e.g., amyotrophic lateral sclerosis, Huntington's disease). In addition, the use of genome editing as a gene therapy is being explored for some diseases, but this has so far only reached clinical testing in the treatment of mucopolysaccharidoses. Based on the large number of planned, ongoing, and completed clinical trials for rare genetic central nervous system diseases, it can be expected that several novel gene therapies will be approved and become available within the near future. Essential for this to happen is the in depth characterization of short- and long-term effects, safety aspects, and pharmacodynamics of the applied gene therapy platforms.

## Introduction

Classically, the majority of medical treatments have been developed for diseases affecting large number of patients and patients with chronic and recurrent treatment needs. Consequently, patients suffering from rare diseases have been left with few or no treatment options. With the advent of gene therapy and other advanced therapies a paradigm shift with more ambitious treatment goals, including disease modification and potential cures, is on the horizon for treatment of rare diseases. Even though a rare disease encompasses few patients, the number of rare diseases amount to more than 6,000 rare diseases, and affect a total of 3.5–5.9% of all people, equating to 263–446 million people globally (Wakap et al., 2020). In addition, it is worth noting that the majority of rare diseases have a genetic and often monogenic origin (Lee et al., 2020). While there is no globally accepted definition of rare disease, there is an overall acceptance of point prevalence setting the threshold in the scientific and regulatory frameworks (Wakap et al., 2020). According to the harmonized standards in the EU regulation on orphan medicinal products, a rare disease affects <50 in 100,000 people, and as defined by the US Food and Drug Administration (FDA) in the Orphan Drug Act, a rare disease affects <200,000 people in the US alone (corresponding at present to approximately 61 in 100,000 people) (Wakap et al., 2020). The average prevalence threshold for the term “rare disease” was calculated as 40 in 100,000 by ISPOR (Rare Disease Special Interest Group) (Richter et al., 2015). Thus, it appears that the overall international consensus is that a *rare disease affects*<*40–60 in 100,000 people*, and this is the definition applied in the present review. The definition, including the patient numbers, is important to drug developers aspiring to enter the fast-track and orphan drug programs for development of treatments for patients with rare diseases which can include additional regulatory support and advising, as well as economic incentives, and market exclusivity. Here, we focus on the current development and prospects of gene therapies for treatment of a subgroup of rare diseases, namely, rare diseases affecting the brain and spinal cord with known genetic etiology.

### A Short Overview of Gene Therapy Development

Already back in the 1970s, it was recognized that gene therapy, replacing or supplementing defective disease-causing DNA with exogenous healthy or beneficial DNA, could hold the promise of offering viable treatment options for human genetic diseases (Friedmann and Roblin, 1972). In the 1980s, the concept formed of using a virus vector for gene transfer into mammalian cells (Williams et al., 1984), and, in 1990, the first approved gene therapy trial took place with viral vector-mediated transfer of the gene encoding the enzyme adenosine deaminase (ADA) in a 4-year-old patient suffering from chromosome X-linked severe combined immunodeficiency (SCID-X1) due to ADA deficiency (Blaese et al., 1995). Hereafter followed a decade of new trials and great optimism, which culminated in two trials with unfortunate outcomes, and a transient halt of further gene therapy trials. In the first case, involving adenovirus (Ad) vector-mediated gene therapy in ornithine transcarbamylase deficiency, unexpected events led to severe vector-associated toxicity, multi-organ failure, and the death of an 18-year-old man (Raper et al., 2003). In the second case, a gamma-retrovirus (γRV) vector-mediated gene therapy encoding for interleukin-2 receptor gamma chain in patients with SCID-X1 was associated with development of genotoxic adverse events and uncontrolled clonal T-cell proliferation in six patients after RV host genome integration and the activation of LIM domain only-2 (LMO2) proto-oncogenes (Hacein-Bey-Abina et al., 2003). Hereafter followed a lock-down period of clinical trials. In the following years, new and safer viral vectors, including a large number of adeno-associated viral (AAV) vectors were discovered (Gao et al., 2005) and introduced to new gene therapy development programs. Recombinant AAVs that are deprived of viral DNA, essentially rendering them a non-replicable protein-based gene transfer carrier, have been favored in the central nervous system (CNS) gene therapy due to their desirable safety profile including low immunogenicity potential and strong neuronal tropism (Hudry and Vandenberghe, 2019). A little more than a decade later, the first gene therapy in Europe, Glybera® (alipogene tiparvovec) for treatment of lipoprotein lipase deficiency, was approved in 2012 (Watanabe et al., 2015). In 2016, the *ex vivo* hematopoietic stem and progenitor cell (HSPC) gene therapy Strimvelis® was approved for treatment of ADA-SCID (Aiuti et al., 2017) and, in 2019, Zynteglo was approved for treatment of beta-thalasemia (Schuessler-Lenz et al., 2020), both in Europe. Subsequently, Luxturna® (voretigene neparvovec), the first gene therapy against inherited eye diseases was approved in the US and Europe in 2017 and 2018, respectively, followed by approval of Zolgensma® (onasemnogene abeparvovec), a gene therapy targeting motor neurons residing in the CNS with axonal projections into the PNS, for treatment of spinal muscular atrophy in US and Europe in 2019 and 2020, respectively (Keeler and Flotte, 2019). The latest addition is the approval of Libmeldy®, an *ex vivo* gene therapy with lentivirus vector (LV)-transduced autologous CD34-positive hematopoetic stem and pluripotent cells (HSPCs) for treatment of metachromatic leukodystrophy, in Europe in 2020 (Bulaklak and Gersbach, 2020).

The current and applicable definitions of human gene therapy from the FDA (*Cellular & Gene Therapy Guidances*, July 20, 2018) and the EU commission (Directive 2001/83/EC, Part IV of Annex I) can be summed up as a biological medicinal product containing recombinant nucleic acid used in or administered to a human to regulate, repair, replace, add, or delete a genetic sequence with the aim to treat or cure diseases. The discipline of gene therapy includes: (1) *in vivo* vector-mediated gene therapy, (2) *ex vivo* cell transduction gene therapy, and (3) genome editing (Brenner et al., 2020). Treatments with antisense oligonucleotides (ASOs) are outside the scope of this review and will only be mentioned briefly when relevant.

### *In vivo* Vector-Mediated Gene Therapy

Generally, there are two types of vectors coming from either viral or non-viral origin, and the viral vector platforms are predominantly based on Ad, AAV, or retro-/lentiviruses due to observed efficacy, safety profile, and regulatory acceptance. The objective of gene transfer is often to compensate for a pathogenic loss-of-function (LoF) mutation by delivery of a functional gene copy or to downregulate the expression of a pathogenic gain-of-toxic-function (GoTF) mutation by delivery of DNA encoding short hairpin RNA (shRNA), small interfering RNA (siRNA), microRNA (miRNA), or antisense RNA (Mitchell et al., 2010; Wang and Gao, 2014). The pharmacokinetics and tissue/cell specificity depend on the selected vector, surface proteins, and cis-acting elements such as promotor elements.

From early on, Ad vectors were applied due to the efficient transduction of dividing and non-dividing cells, high transgene capacity, and low insertional mutagenesis rate (Gray et al., 2010). However, despite development of newer and improved generations of Ad vectors, challenges persist with pre-existing viral immunity, induction of strong innate immune responses toward capsid proteins, and adaptive immune response to viral and transgene products, which has led to Ad-based vectors no longer being preferred in trials targeting CNS disorders (Sing et al., 2018; Goswami et al., 2019). In other therapeutic areas where the associated challenges are less of a problem Ad vectors are still applied, e.g., in vaccines and oncolytic therapies.

Recombinant AAV vectors have found particular use in treatment strategies for CNS diseases (Mendell et al., 2021). AAV vectors are versatile and induce expression in both dividing and non-dividing cells and remain predominantly as single- or double-stranded DNA within the cell nucleus in episomal form (Salganik et al., 2015), although, *in vivo* and *in vitro* characterizations have suggested an integrative potential for wild-type AAV into a specific site in chromosome 19 in the human (Kotin et al., 1990, 1991). AAVs and their simple DNA genomes are well-studied, and AAV-based vectors have been shown to deliver long-term transgene expression, which has been documented up to 10 years in humans and up to 15 years in non-human primates after administration (Sehara et al., 2017; Chu et al., 2020). Several different AAV serotypes have been discovered, which differ by their specific tropism and tissue specificity linked to the diverse surface capsid proteins they express. These capsids have been discovered by (1) vectorization from natural isolates, (2) from rational designs using pre-existing capsids (Chen et al., 2009), (3) directed evolution using interative selection of mutated capsids, e.g., AAV2.7m8 (Dalkara et al., 2013), AAVPHP.B (Deverman et al., 2016), and AAV-F (Hanlon et al., 2019), (4) and by *in silico* approaches using computation tools to design novel synthetic capsids (Wang et al., 2019). So far, the approved AAV gene therapies, such as Glybera® and Luxturna®, are derived from naturally occurring variants (AAV capsid serotype 1 and 2, respectively). Currently, AAV vectors are regarded the least immunogenic and with less vector-associated toxicity, which make them preferred for many CNS diseases. Nonetheless, important safety concerns still need to be tackled, especially regarding genome integration issues, long-term sustained safety (Nguyen et al., 2021), and risk of high-dosing induced toxicity (Hinderer et al., 2018).

The retroviridae family has provided the simple γRV and the more complex lentiviruses, which have both been applied for gene therapy. Whereas the γRV was used earlier, the field has moved to prefer the lentivirus, and especially the HIV-1 virus as vector platform. LVs possess desirable characteristics, including genome integration for persistent long-term transgene expression in both postmitotic and quiescent cells (Naldini et al., 1996), low immunogenic potential (Abordo-Adesida et al., 2005), and relatively large transgene cassette capacity enabling expression of multiple genes from a single vector construct (Zhu et al., 2001; Tian and Andreadis, 2009). In contrast to the gamma-retroviral vectors, the LVs do not integrate into the genome within the proximity of oncogene transcriptional start sites, making them much less prone to oncogenic risk, and therefore they are regarded as much safer (Schröder et al., 2002; Cattoglio et al., 2007). In addition, lentivirus vectors have been modified to minimize the risk of host genome integration or to direct the insertional mutagenesis into heterochromatin regions (not affecting gene activation or silencing), for safe and stable transduction of non-dividing cells or transient transduction in actively dividing cells (Lentz et al., 2012). The development of more efficient and safer vectors over the years has resulted in LVs, which are self-inactivating and replication-incompetent (Zufferey et al., 1998). Using pseudotyping with glycoproteins have enabled specific tropisms and tissue-specificity, and have facilitated specific transduction to the retina and HSPCs (Duisit et al., 2002; MacKenzie et al., 2002). LVs have been tested in many successful clinical trials, and have become a preferred tool in particular in *ex vivo* gene therapy strategies for treating genetic diseases (see below).

Non-viral vectors with different transgene encapsulations exist, but despite that the first lipid nanoparticle-based RNA interference (RNAi) therapeutic drug was approved for treatment in 2018 (Kimura and Harashima, 2020), the viral vector-mediated gene transfers are still the preferred choice for gene therapies in the CNS. Finally, new genetic tools using *in vivo* gene therapy such as chemogenetics and optogenetics (Ingusci et al., 2019), have been developed as useful tools for basic scientific research, but could also refine gene therapy approaches to control neuronal activation for rare genetic disorders in the CNS in the future.

### *Ex vivo* Cell Transduction Gene Therapy

*Ex vivo* HSPC transduction gene therapy (HSPC-GT) has played a central role in the development of gene therapies, as mentioned above, with the successful treatment of ADA-SCID with γRV-based vectors (Blaese et al., 1995; Aiuti et al., 2009) and later with the unfortunate occurrence of genotoxic events in SCID-X1 patients disrupted the immediate success (Hacein-Bey-Abina et al., 2003). This led to the increased usage of LVs derived from the human immunodeficiency virus (HIV), which are believed to possess a safer integration profile and much lower risk of insertional mutagenesis (Tucci et al., 2021). In general, gene transfer into autologous HSPCs has the potential to provide permanent therapeutic gene expression as a selective treatment in monogenic inherited disorders, and can be exploited as cell vehicles to deliver proteins into the circulation and tissues, including the CNS (Tucci et al., 2021). Briefly, patients' own cells are collected and stem cells are isolated to be mixed and transduced with a viral vector encoding a desired therapeutic gene. The transduced stem cells are later re-infused in the patient engrafted in the tissue, aiming at restoring a healthy phenotype (Penati et al., 2017). Thus, applying autologous HSPCs has become a viable treatment option for some patients with inborn errors of metabolism, providing enduring effect while reducing the risk of allogenic treatment-related toxicities and development of graft-vs.-host-disease as seen with donor HSPCs from healthy individuals (Morgan et al., 2017). HSPCs have lifelong ability to self-renew and to differentiate into specific cell types which make them an attractive target for gene therapy. Although HSPCs are not fully characterized, the expression of a surface glycoprotein, CD34 (CD34+), and lack of another one, CD38 (CD38-), allow for selection and purification when grown *ex vivo* after collecting from the patients (Hossle et al., 2002). Furthermore, the CD34+/CD38- HSPCs can be subdivided depending on the presence or absence of CD90 and CD45RA glycoproteins which can aid in selecting the optimal population for HSPC-GT (Majeti et al., 2007). LVs derived from HIV are the preferred vectors due to superior safety and efficacy parameters, including self-inactivating configuration to minimize the risk of producing replication-competent lentiviral particles and with a safer integration profile as compared to other retroviral vectors (Tucci et al., 2021). The focus has, so far, mainly been on application in monogenic disorders affecting the lysosomal and peroxisomal metabolic activity impairing CNS functions, which leads to oxidative stress, local inflammation, microglial activation, progressive demyelination, and axonal degeneration (Tucci et al., 2021). The first clinical application of lentiviral-based gene therapy was for treatment of inherited metabolic disorders including X-linked adrenoleukodystrophy (Eichler et al., 2017), metachromatic leukodystrophy (Rosenberg et al., 2016), and mucopolysaccharidoses (Kinsella et al., 2020), with the aim of increasing enzyme bioavailability and brain entry (Begley et al., 2008) for correction of the neuropathological phenotype (see below in the disease sections for more details). Several projects appear promising based on pre-clinical and clinical data, however, challenges remain including the validation of long-term sustained efficacy and safety profile in patients who received γRV- or LV-based HSPC-GT *ex vivo*.

### Genome Editing

Genome editing enables insertion, deletion, or replacement of nucleotides, but also modulation of gene expression and epigenetic editing (Duarte and Déglon, 2020). In monogenetic CNS diseases, a disease-causing mutation leading to either LoF or GoTF can be corrected by targeted editing of the specific mutation to restore a healthy phenotype. The available editing tools include zinc finger nucleases (ZFNs), transcription activator-like effector nucleases (TALENs), or the CRISPR/Cas systems (Goswami et al., 2019; Poletto et al., 2020).

ZFNs are eukaryotic specific DNA-binding domains consisting of two anti-parallel beta-sheets and one alfa-helix, binding to triplet DNA sequences, and with intrinsic nuclease activity to open up DNA strands (Miller et al., 1985; Pavletich and Pabo, 1991). Realizing that the early modular assembly ZFNs were too error proned (Ramirez et al., 2008), the development has moved toward selection-guided assembly ZFNs (Greisman and Pabo, 1997; Cornu et al., 2008), and creation of synthetic ZFN oligomers displaying higher affinity and specificity toward larger multiple triplet basepair sequences (Urnov et al., 2010). This increase in combinatorial opportunities means that it is now possible to select ZFNs targeting almost any thinkable DNA sequence. TALENs are molecularly programmable nucleases inside of a central array of 33-35 amino acid motifs, recognizing single bases (Boch et al., 2009; Miller et al., 2011; Zhang et al., 2011). However, the cloning and protein engineering work for ZFNs and TALENs is complex and requires extensive expertise in molecular biology to take advantage of those techniques, which has limited their general distribution and application. CRISPR/Cas are based on RNA-guided nucleases and DNA-binding properties which are easily modulated by a short RNA sequence (Fineran and Charpentier, 2012; Wiedenheft et al., 2012). They can be grouped into two main classes according to their nuclease effectors (Makarova et al., 2015, 2020): The class 1 systems (types I, III, and IV) involve a large complex of several effector proteins, and the class 2 systems (types II, V, and VI) use a single Cas protein to mediate the recognition and cleavage of foreign nucleic acids. The class 2 systems are most widely used because of their simple structure, and the type II and type V CRISPR/Cas ribonucleoprotein complexes recognize specific DNA sequences through RNA-DNA base pairing and induce a double strand break. The host cell responds to this break by a mechanism known as homology-directed repair where donor DNA is offered as a template for the repair, thereby allowing gene editing and repair based on a healthy DNA template (Karimian et al., 2018; Yeh et al., 2019). Recently, the CRISPR/Cas-based genome editing has prevailed over the ZFNs and TALENs since they are easier to engineer to recognize unique sequences. The DNA-binding specificity of ZFNs and TALENs is dependent on protein-DNA interactions whereas the CRISPR/Cas is provided by the sgRNAs, which are simpler and less expensive to design (Duarte and Déglon, 2020).

Despite making progress in pre-clinical studies (Lubroth et al., 2021), only a few *in vivo* genome editing approaches utilizing ZFNs in CNS have reached clinical trials. Nonetheless, the potential of expanding into CNS diseases is clear, and there should be a great interest from the pharmaceutical industry to advance more *in vivo* genome editing into clinical stages within brain and spinal cord diseases. For this to become a reality the identified challenges and safety concerns have to be solved, especially related to immunogenicity induced by the genome editing tools (Shim et al., 2017) as well as better characterization and control of on- and off-target modifications (Mills et al., 2003).

### Delivery Strategies for Gene Therapy to the Brain and Spinal Cord

Delivery of genetic material is an important issue since accessibility of systemically administered treatments targeting the CNS has long been complicated by the tightly regulated blood-brain barrier (BBB) that controls passage into the CNS (Kimura and Harashima, 2020). Direct intracerebral injection into the parenchyma, although highly invasive, is one way to circumvent this problem, which also ensures a direct match between treatment delivery and the targeted region. This method could be preferred when the target is a defined and limited area of the brain or when targeting deeper brain structures in humans such as the thalamus or putamen (Hocquemiller et al., 2016; Taghain et al., 2020). Intracerebral administration into specific brain regions will typically be associated with the administration of lower numbers of viral genomes compared to those required for systemic administration which limits the risk of toxicity. A rat study preparing for human trials for Parkinson's disease found that the no observed adverse effect level (NOAEL) dose was 6.8 × 10^8^ viral genomes of an AAV2 vector encoding glial-derived neurotrophic factor administered as a single intracerebral dose into the striatum (Terse et al., 2021). Nonetheless, toxicity and spread of transgene expression in different regions and cell types may vary extensively depending on the titer/volume injected and serotype of vectors used (Peters et al., 2021).

If it is necessary to deliver treatment to larger parts of the brain or spinal cord, it is possible to apply multiple injection sites and/or viral vectors with a larger degree of spread and retrograde transport along neuronal processes (Kimura and Harashima, 2020). Another strategy for achieving a wide spread in the brain and spinal cord could be delivered through the CSF, by intracerebroventricular (ICV), intracisterna magna, or intrathecal injections (Hocquemiller et al., 2016; Taghain et al., 2020). Intrathecal injections are conveniently achieved by lumbar puncture and achieve extensive spinal cord transduction whereas administration into the cisterna magna delivers the drug closer to the targeted brain areas and has shown transduction in the spinal cord as well as brain (Taghain et al., 2020). Studies have demonstrated that AAV vectors, when injected into the cerebrospinal fluid, deliver genes throughout the brain and spinal cord in non-human primates (Bey et al., 2020).

As the cell and tissue tropism of different AAV serotypes became better understood, it also became apparent that some serotypes are better than others at reaching the CNS after systemic administration, for example, AAV9, AAVrh8, AAVrh10, and AAVHSC15 can cross the BBB after intravenous (IV) administration, resulting in widespread transduction of the CNS and peripheral organs through a less invasive procedure (Yang et al., 2014; Ellsworth et al., 2019; Belur et al., 2020). Intravascular administration is the current delivery method for Zolgensma®, approved for treatment of SMA1, as discussed later, which utilizes the AAV9 vector capabilities of crossing the BBB (Chen, 2020). In addition, new AAV subtypes, such as AAV-PHP.B, show up to 40 times higher efficiency at transducing neurons and astrocytes when compared to IV-injections of AAV9 (Liu et al., 2021). However, it appears that AAV-PHP.B expression is species dependent, expressing at much lower levels in BALB/cJ mice and non-human primates than in C57BL/6J mice, and toxicity observed in non-human primates indicate that its usefulness in humans may consequently be limited (Hordeaux et al., 2018, 2019).

## Rare Diseases and Prospects of Utilizing Gene Therapies

We here provide an overview of the developmental progress for novel gene therapeutic treatments for rare genetic diseases in the brain and spinal cord, with a special focus on clinical development. For diseases which have not reached clinical testing, we seek to describe the current status and near-future prospects. Overviews are given of current gene therapy clinical trials from https://clinicaltrials.com (last search on the 1st of May 2021) for the therapeutic areas in Table 1 and for the individual diseases and trials in Table 2.

**Table 1:** Overview of clinical gene therapy trials for groups of rare genetic diseases affecting the brain and spinal cord found on https://clinicaltrials.gov 1st of May 2021.

**Group**	**Disease**	**Prevalence per 100,000**	**Number of Trials**	**Clinical Trial Phase Reached**
Neurodegenerative Disorders	Spinal Muscular Atrophy (SMA)	10	9	Gene therapy market approval by FDA and EMA (Zolgensma®)
	Multiple System Atrophy (MSA)	2	1	Gene therapy trial planned, not yet recruiting
	Amyotrophic Lateral Sclerosis (ALS)	5	1	Compassionate-use study in two patients
	Huntington's Disease (HD)	3	1	Phase 1/2 trial
Neurodevelopemental Disorders	Rett Syndrome (RTT)	10–12	0	No clinical trials with gene therapy
	Genetic Syndromes (e.g., Dravet, Lennox-Gastaut, West, and Angelman synromes)	3–15	0	No clinical trials with gene therapy
Lysosomal Storage Diseases	Batten Disease (CLN, Neuronal Ceroid Lipofuscinoses)	2–4	6	Phase 1/2 trials and LTFU
	Krabbe Disease (KD)	1	2	Phase 1/2 trials
	Metachromatic Leukodystrophy (MLD)	1–2	6	*Ex vivo* autologous hematopoietic stem cell gene therapy approved by EMA (Libmeldy®)
	Mucopolysaccharidoses (MPS)	4	19	Phase 2/3 trials
Neurometabolic Disorders	Canavan Disease (CD)	<16	3	Phase 1/2 trials
	Niemann-Pick Disease	5	0	No clinical trials with gene therapy
	X-linked Adrenoleukodystrophy (ALD)	7	5	Phase 2/3 trial
	Phenylketonuria (PKU)	10	3	Phase 1/2 trials
	Gangliosidoses (GM1/2)	1	5	Phase 1/2 trials

**Table 2:** Overview of clinical gene therapy trials for rare genetic diseases affecting the brain and spinal cord found on https://clinicaltrials.gov 1st of May 2021.

**Disease (OMIM)**	**Intervention and dose**	**Route of administration**	**NCT number**	**Phase**	**Participants**	**Ages eligible**	**Sponsors**	**Study period**	**Status (May 2021)**
SMA (600354)	scAAV9-SMN (AVXS-101) onasemnogene abeparvovec-xioi (6.7 ×10^13^ – 2.0 ×10^14^ vg/kg)	IV	NCT02122952	1/2	15	≤ 6 months	AveXis	05/2014–12/2017	Completed
	scAAV9-SMN (AVXS-101) onasemnogene abeparvovec-xioi (1.1 ×10^14^ vg/kg)	IV	NCT03306277	3	22	≤ 180 days	AveXis	10/2017–11/2019	Completed
	scAAV9-SMN (AVXS-101) onasemnogene abeparvovec-xioi (6.0 ×10^13^ – 1.2 ×10^14^ – 2.4 ×10^14^ vg)	IT	NCT03381729	1/2	51	6–60 months	Novartis	12/2017–06/2021	Suspended
	scAAV9-SMN (AVXS-101) onasemnogene abeparvovec-xioi (LTFU, no additional dosing)	IV	NCT03421977	LTFU	13	Child- to adulthood	Novartis	09/2017–13/2033	Active not recruiting
	scAAV9-SMN (AVXS-101) onasemnogene abeparvovec-xioi (1.1 ×10^14^ vg/kg)	IV	NCT03461289	3	33	≤ 180 days	AveXis	08/2018–09/2020	Completed
	scAAV9-SMN (AVXS-101) onasemnogene abeparvovec-xioi (1.1 ×10^14^ vg/kg)	IV	NCT03505099	3	30	≤ 42 days	Novartis	05/2018–07/2021	Active not recruiting
	scAAV9-SMN (AVXS-101) onasemnogene abeparvovec-xioi (dose unknown)	IV	NCT03837184	3	2	≤ 6 months	Novartis	05/2019–06/2021	Active not recruiting
	scAAV9-SMN (AVXS-101) onasemnogene abeparvovec-xioi (LTFU, no additional dosing)	IV/IT	NCT04042025	LTFU	308	Child- to adulthood	Novartis	02/2020–12/2035	Enrolling by invitation
MSA (146500)	AAV2-GDNF (dose unknown)	IC (putamen)	NCT04680065	1	9	35–75 years	Brain Neurotherapy Bio/Asklepios Biopharmaceutical	01/2021–01/2024	Not yet recruiting
ALS	AAVrh10-miR-SOD1 (4.2 ×10^14^ vg)	IT	n/a	n/a	2	22 and 56 years	Univ. of Massachusetts Medical School / Massachusetts General Hospital / Harvard Medical School	07/2017–05/2020	Completed
HD (143100)	AAV5-miHTT (AMT-130) (6 ×10^12^ – 6 ×10^13^ gc)	IC (striatum)	NCT04120493	1/2	26	25–65 years	UniQure Biopharma	09/2019–05/2026	Recruiting
BD (CLN2) (204500)	AAV2-CUhCLN2 (3 ×10^12^ particle units)	IC	NCT00151216	1	10	3–18 years	Weill Medical College of Cornell University	06/2004–06/2019	Completed
	AAVrh10-CUhCLN2 (2.85 ×10^11^ – 9 ×10^11^ gc)	IC	NCT01161576	1	12	2–18 years	Weill Medical College of Cornell University	08/2010–12/2020	Completed
	AAVrh10-CUhCLN2 (2.85 ×10^11^ – 9 ×10^11^ gc)	IC	NCT01414985	1/2	8	3–18 years	Weill Medical College of Cornell University	04/2010–02/2017	Completed
BD (CLN3) (204200)	AAV9-CLN3 (AT-GTX-502) (6 ×10^13^ – 1.2 ×10^14^ vg)	IT	NCT03770572	1/2	7	3–10 years	Amicus Therapeutics	11/2018–09/2023	Active not recruiting
BD (CLN6) (606725)	AAV9-CLN6 (AT-GTX-501) (dose unknown)	IT	NCT02725580	1/2	13	≥1 year	Amicus Therapeutics	03/2016–11/2021	Active not recruiting
	AAV9-CLN6 (AT-GTX-501) (LTFU, no additional dosing)	IT	NCT04273243	LTFU	13	≥1 year	Amicus Therapeutics	01/2020–01/2035	Recruiting
KD (245200)	AAVrh10-hGALC (FBX-101) (dose unknown)	IV	NCT04693598	1/2	6	≤ 12 months	Forge Biologics	01/2021–04/2023	Not yet recruiting
	AAVhu68-GALC (PBKR03) (1.5 ×10^11^ – 5.0 ×10^11^ gc/g brain mass)	ICM	NCT04771416	1/2	24	1–9 months	Passage Bio	06/2021 - 01/2030	Not yet recruiting
MLD (250100)	LV-ARSA (OTL-200) (*ex vivo*) CD34+ HSPC (dose unknown)	IV	NCT01560182	1/2	20	≤ 7 years	Orchard Therapeutics. Telethon	04/2010–04/2023	Active not recruiting
	AAVrh10-cuARSA (1 ×10^12^ – 4 ×10^12^ vg)	IC	NCT01801709	1/2	5	6 months−5 years	Institut National de la Santé et de la Recherche Médicale	03/2013–04/2019	Active not recruiting
	LV-ARSA (*ex vivo*) CD34+ HSPC (2 ×10^6^ – 20 ×10^6^ per kg)	IV	NCT02559830	1/2	50	2–45 years	Shenzhen Second People's Hospital	01/2015–10/2025	Recruiting
	LV-ARSA (OTL-200) (*ex vivo*) CD34+ HSPC (dose unknown)	IV	NCT03392987	2	10	≤ 6 years	Orchard Therapeutics, Telethon	01/2018–08/2028	Active not recruiting
	LV-ARSA (1 ×10^9^ – 2 ×10^9^ moi/ml per site)	IC	NCT03725670	1/2	10	≥1 month	Shenzhen Geno-Immune Medical Institute	10/2018–11/2020	Recruiting
	LV-ARSA (OTL-200) (*ex vivo*) CD34+ HSPC (dose unknown)	IV	NCT04283227	3	6	Child- to adulthood	Orchard Therapeutics, Telethon	12/2020–01/2032	Recruiting
MPS I (607015)	Various, venipuncture (LTFU, no additional dosing)	Various	NCT00695279	LTFU	100	Child- to adulthood	St. Jude Children's Research Hospital	01/2007–12/2036	Recruiting
	AAV6-ZFN-IDUA (SB-318) (dose unknown)	IV	NCT02702115	1/2	3	≥5 years	Sangamo Therapeutics	05/2017–01/2022	Active not recruiting
	LV-IDUA (*ex vivo*) CD34+ HSPC (target dose 8 ×10^6^ CD34+ cells/kg)	IV	NCT03488394	1/2	8	≤ 11 years	IRCCS San Raffaele	05/2018–01/2023	Recruiting
	AAV9-IDUA (RGX-111) (1 ×10^10^ – 5 ×10^10^ gc/g brain mass)	IC	NCT03580083	1/2	5	≥4 months	Regenxbio	04/2019–07/2023	Recruiting
	AAV6-ZFN-IDUA (SB-318) (LTFU, no additional dosing)	IV	NCT04628871	LTFU	13	≥18 years	Sangamo Therapeutics	11/2020–01/2030	Enrolling by invitation
MPS II (309900)	AAV6-ZFN-IDS (SB-913) (dose unknown)	IV	NCT03041324	1/2	9	≥5 years	Sangamo Therapeutics	05/2017–02/2022	Active not recruiting
	AAV9-IDS (RGX-121) (1.3 ×10^10^ – 2.0 ×10^11^ gc/g brain mass)	IC	NCT03566043	1/2	12	4 months−5 years	Regenxbio	09/2018–12/2023	Recruiting
	AAV9-IDS (RGX-121) (6.5 ×10^10^ gc/g brain mass)	ICM/ICV	NCT04571970	1/2	6	5–17 years	Regenxbio	02/2021–06/2023	Recruiting
	AAV9-IDS (RGX-121) (LTFU, no additional dosing)	ICM/ICV	NCT04597385	LTFU	12	≥28 months	Regenxbio	03/2021–09/2025	Enrolling by invitation
	AAV6-ZFN-IDS (SB-913) (LTFU, no additional dosing)	IV	NCT04628871	LTFU	13	≥18 years	Sangamo Therapeutics	11/2020–01/2030	Enrolling by invitation
MPS IIIA (252900)	AAVrh10-hSGSH-IRES-SUMF1 (SAF-301) (dose unknown)	IC	NCT01474343	1/2	4	18 months−6 years	Lysogene	08/2011–05/2013	Completed
	AAVrh10-hSGSH-IRES-SUMF1 (SAF-301) (dose unknown)	IC	NCT02053064	1/2	4	Child- to adulthood	Lysogene	05/2013–06/2017	Completed
	scAAV9.U1a.hSGSH (ABO-102) (0.5 ×10^13^ – 3 ×10^13^ vg/kg)	IV	NCT02716246	1/2	22	≥6 months	Abeona Therapeutics	03/2016–12/2022	Recruiting
	AAVrh10-hSGSH (LYS-SAF302) (dose unknown)	IC	NCT03612869	2/3	20	≥6 months	Lysogene	12/2018–03/2022	Active not recruiting
	scAAV9.U1a.hSGSH (ABO-102) (3 ×10^13^ vg/kg)	IV	NCT04088734	1/2	12	Child- to adulthood	Abeona Therapeutics	10/2019–12/2023	Recruiting
	LV-SGSH (*ex vivo*) CD34+ HSPC (dose unknown)	IV	NCT04201405	1/2	5	3–24 months	University of Manchester, Orchard Therapeutics	01/2020–10/2024	Recruiting
	scAAV9.U1a.hSGSH (ABO-102) (LTFU, no additional dosing)	IV	NCT04360265	LTFU	50	Child- to adulthood	Abeona Therapeutics	10/2020–12/2025	Recruiting
MPS IIIB (252920)	AAV5-hNAGLU (4 ×10^12^ vg)	IC	NCT03300453	1/2	4	18–60 months	UniQure Biopharma	10/2013–11/2019	Completed
	AAV9-hNAGLU (2 ×10^13^ – 1 ×10^14^ vg/kg)	IV	NCT03315182	1/2	15	Child- to adulthood	Abeona Therapeutics	10/2017–10/2022	Recruiting
CD (271900)	AAV2-hASPA (9 ×10^11^ vg)	IC	n/a	1	13	4–83 months	n/a	2001–2005	Completed
	AAV2-hASPA (LTFU, no additional dosing)	IC	n/a	LTFU	13	4–83 months	n/a	2006–2011	Completed
	AAV-oligo001-ASPA (3.7 ×10^13^ vg)	ICV	NCT04833907	1/2	24	3–60 months	CureRare Disease LLC	04/2021–03/2024	Recruiting
ALD (300100)	LV-ALD (*ex vivo*) CD34+ HSPC (dose unknown)	IV	NCT01896102	2/3	32	≤ 17 years	Bluebird bio	08/2013–05/2021	Active not recruiting
	LV-ABCD1 (*ex vivo*) CD34+ HSPC (2 ×10^6^ – 2 ×10^7^ CD34+ cells/kg)	Infusion	NCT02559830	1/2	50	2–45 years	Shenzhen Second People's Hospital	01/2015–10/2025	Recruiting
	LV-ALD (*ex vivo*) CD34+ HSPC (LTFU, no additional dosing)	IV	NCT02698579	LTFU	60	Child- to adulthood	Bluebird bio	01/2016–05/2037	Enrolling by invitation
	LV-ABCD1 (1 ×10^9^ – 2 ×10^9^ moi/ml per site)	IC	NCT03727555	1/2	10	Child- to adulthood	Shenzhen Geno-Immune Medical Institute	10/2018–10/2020	Recruiting
	LV-ALD (*ex vivo*) CD34+ HSPC (dose unknown)	IV	NCT03852498	3	35	≤ 17 years	Bluebird Bio	01/2019–02/2024	Recruiting
PKU (261600)	AAV.HSC15-PAH (HMI-102) (dose unknown)	IV	NCT03952156	1/2	21	18–55 years	Homology Medicines	06/2019–09/2021	Recruiting
	AAV.HSC15-hPAH (HMI-102) (LTFU, no additional dosing)	IV	NCT04348708	LTFU	21	18–55 years	Homology Medicines	08/2020–12/2026	Enrolling by invitation
	AAV5-PAH (BMN 307) (dose unknown)	IV	NCT04480567	1/2	100	≥15 years	BioMarin Pharmaceutical	09/2020–12/2027	Recruiting
GM1 (230500)	AAV9-GLB1 (1.5 ×10^13^ – 4.5 ×10^13^ vg/kg)	IV	NCT03952637	1/2	45	6 months−12 years	National Human Genome Research Institute, Axovant Sciences	08/2019–05/2024	Recruiting
	AAVrh10-GLB1 (LYS-GM101) (8 ×10^12^ vg/kg)	ICM	NCT04273269	1/2	16	≤ 3 years	Lysogene	03/2021–06/2025	Active Not recruiting
	AAVhu68-GLB1 (PBGM01) (3.3 ×10^10^ – 1.1 ×10^11^ gc/g brain mass)	ICM	NCT04713475	1/2	20	4–36 months	Passage Bio	02/2021–02/2029	Recruiting
GM2 (230600)	AAVrh8-HEXA/AAVrh8-HEXB (AXO-AAV-GM2) (dose unknown)	IC/ICM/IT	NCT04669535	1	18	6 months−12 years	Sio Gene Therapies	01/2021–06/2028	Recruiting
	AAV9-HEXA/HEXB (TSHA-101) (dose unknown)	IT	NCT04798235	1/2	6	≤ 12 months	Taysha Gene Therapies	03/2021–03/2027	Recruiting

*AAV, adeno-associated virus; ALD, X-linked adrenoleukodystrophy; ALS, amyotrophic lateral sclerosis; ARSA, arylsulfatase; BD, Batten disease; CD, Canavan disease; CLN, ceroid lipofuscinosis neuronal; GALC, galactosylceramidase; gc, genome copies; GDNF, glial cell line-derived neurotrophic factor; GLB1, galactosidase beta 1; GM, gangliosidosis; HD, Huntington's disease; HSPC, hematopoietic stem and progenitor cells; HTT, huntingtin; IC, intracerebral; ICM, intracisterna magna; ICV, intracerebroventricular; IDS, iduronate-2-sulfatase; IDUA, α-L-iduronidase; IT, intrathecal; IV, intravenous; KD, Krabbe disease; LTFU, long-term follow-up; LV, lentivirus; MLD, metachromatic leukodystrophy; moi, multiplicity of infection; MPS, mucopolysaccharidoses; MSA, multiple system atrophy; n/a, not applicable; NAGLU, alpha-N-acetylglucosaminidase; PKU, phenylketonuria; RTT, Rett syndrome; SGSH, N-sulfoglucosamine sulfohydrolase; SMA, spinal muscular atrophy; SMN, survival motor neuron; SUMF1, sulfatase-modifying factor 1; vg, vector genomes; ZFN, zinc finger nuclease*.

## Neurodegenerative Disorders

### Spinal Muscular Atrophy (SMA)

SMA is characterized by degeneration of spinal cord alpha motor neurons resulting in muscular wasting. The disease impairs the patient's ability to walk, speak and breathe (Pattali et al., 2019). It affects approximately 10 in 100,000 newborns and is the most common monogenic disease leading to death in infants (Darras, 2015; Chen, 2020). SMA is the result of a LoF mutation in the *survival of motor-neuron 1 (SMN1)* gene. *SMN1* encodes a protein essential for survival of the alpha motor neurons. The function of the protein is not yet completely understood (Kariyawasam et al., 2018). The human genome has a similar gene, *survival of motor-neuron 2 (SMN2)*, which exists in multiple copies in the genome and is different from *SMN1* only by a few nucleotides, notably a nucleotide variant in exon 7. This variant in *SMN2* leads to exclusion of exon 7, resulting in an unstable protein. It is estimated that the protein production resulting from *SMN2* creates 90% truncated proteins (without exon 7) and generates 10% normal but still unstable SMN proteins. These proteins can partially compensate for the loss of *SMN1*, and it is argued that *SMN2* copy number in the genome determines phenotypic severity (Pattali et al., 2019; Chen, 2020). SMA is classified in different clinical phenotypes based on age at onset of symptoms, the level of motor functions achieved, and number of *SMN2* genes. Type 0 (1 *SMN2* copy): with onset *in utero*, dependent of mechanical ventilation at birth, and survival is usually below 6 months of age; type 1 (Werdnig Hoffman disease) (1-3 *SMN2* copies): onset before 6 months of age, with positure, respiratory, and feeding support required, and expected life expectancy below 2 years of age; type 2 (Dubowith disease) (2-4 *SMN2* copies): onset at 7–18 months, with inability to walk, need for respiratory and feeding support, and with life expectancy to reach adulthood; type 3 (Kugelberg-Welander disease) (3-4 *SMN2* copies): onset at 18 months, initially with ability to stand and walk, however, it is not retained, and with normal life expectancy; type 4 (4 *SMN2* copies): late onset in adulthood and with mild symptoms and normal life expectancy (Munsat and Davies, 1992; Wang et al., 2007). Patients suffering from SMA type 1 (SMA1), the most common form accounting for approximately 60% of all cases, show symptoms including hypotonia, motor delays, and breathing difficulties. The primary cause of death is respiratory failure as a result of muscle weakness. Median survival for SMA1 is estimated at 13.5 months (Rao et al., 2018).

Until recently, the only available medical care for patients suffering from SMA was supportive. In 2016 and 2017, FDA and EMA, respectively, approved nusinersen (Spinraza®), an ASO up-regulating full-length SMN2 transcription which leads to both symptom reduction and halted disease progression (Chen, 2020). Two phase 3, randomized, double-blind trials (CHERISH, NCT02292537; ENDEAR, NCT02193074) have confirmed the efficacy of nusinersen with a 47% reduction in risk of death or permanent ventilation and a favorable safety profile (Finkel et al., 2017; Kariyawasam et al., 2018; Mercuri et al., 2018). Soon after, the first gene therapy for SMA, onasemnogene abeparvovec (AVXS-101) developed by AveXis (acquired by Novartis Pharmaceuticals), was approved under the brand name Zolgensma® by the FDA and EMA in 2019 and 2020, respectively. AVXS-101 is an SMN1 gene replacement therapy delivered by a self-complementary AAV9 (scAAV9) virus that is able to cross the BBB. It has a constitutively active promotor providing persistent expression of SMN1 protein. The efficacy and safety of Zolgensma® (onasemnogene abeparvovec-xioi) have been established in three clinical studies (START, NCT02122952; STR1VE, NCT03306277; and SPR1NT; NCT03505099) and one long-term follow-up study (NCT03421977). Results have been published from the START study, an open-label, dose-escalation, phase 1/2A trial evaluating safety and efficacy of IV delivery of AVXS-101 as a treatment for SMA1 in 15 participants aged up to 6 months and compared with historical controls (Mendell et al., 2017; Al-Zaidy et al., 2019a,b; Lowes et al., 2019). All patients had SMA1, homozygous *SMN1* exon 7 deletions, and two copies of *SMN2*. Significant improvements were reported after AVXS-101 treatment, with all patients surviving past the age of 20 months without requiring permanent ventilation compared to just 8% in the historic cohort (Mendell et al., 2017). In addition, a rapid increase from baseline in the motor function score followed in the high-dose (therapeutic dose) cohort as compared with a decline in this score in the historical control cohort, indicating that of the 12 patients who had received the high dose, 11 sat unassisted, 9 rolled over, 11 fed orally and could speak, and 2 walked independently. During the 24 months follow-up period, the AVXS-101 treated patients spent less time hospitalized with lower number of admissions and length of stay as compared to historical controls (Al-Zaidy et al., 2019a). End-of-study analysis demonstrated that AVXS-101 treatment substantially improved permanent ventilation-free survival, and significantly improved motor function and motor milestone achievement in infants with SMA1 as compared with outcomes observed in the history cohort (Al-Zaidy et al., 2019b). *Post-hoc* analysis revealed that the biggest motor improvements were obtained in infants with SMA1 treated at an early age highlighting the importance of newborn screening and early treatment (Lowes et al., 2019). Therefore, the SPR1NT, a phase 3, open-label, single-arm study was conducted with one-time IV dosing of AVXS-101 in 30 infants up to 42 days of age with genetically diagnosed and pre-symptomatic SMA1 with 1-3 *SMN2* copies (most commonly 2). Results remain to be posted. Moreover, three open-label, single-arm, single-dose, phase 3 trials were conducted in the US (NCT03306277; STR1VE), Europe (NCT03461289; STRIVE-EU), and Asia (NCT03837184) after one-time IV infusion of AVXS-101. In 2021, results were published from the STR1VE study (NCT03306277) in 22 patients younger than 6 months with SMA with biallelic *SMN1* mutations (deletion or point mutations) and one or two copies of *SMN2*, and compared to untreated patients from pediatric neuromuscular clinical research dataset (Day et al., 2021). At the 18 month of age study visit, in patients treated with AVXS-101, 59% achieved functional independent sitting (0% in the control group) and 91% survived free from permanent ventilation at age 14 months (vs. 26% in the control group). The most frequently reported serious adverse events were bronchiolitis, pneumonia, respiratory distress, and respiratory syncytial virus bronchiolitis, and three serious adverse events were related or possibly related to the treatment (two patients had elevated hepatic aminotransferases, and one had hydrocephalus). Long-term benefits and risks await to be determined. The favorable benefit–risk profile suggests that AVXS-101 could provide new hope for treatment of future patients with infantile-onset SMA1. An open-label, dose-escalation, phase 1/2A trial (STRONG; NCT03381729) evaluating safety and efficacy after intrathecal delivery of AVXS-101 (also called OAV-101 in intrathecal administration) as a treatment for SMA with 2 copies of *SMN2* and deletion of *SMN1* in 51 participants aged 6–60 months is ongoing and has recently (August 2021) been allowed by the FDA to resume after nearly a 2 year suspension due to safety concerns. Interim data published from the STRONG study imply that intrathecal administration is feasible, well-tolerated and improve motor funtions in patients with SMA1 (non-ambulatory) and SMA2 patients (Finkel et al., 2019, 2020). Two ongoing phase 4 long-term follow-up trials (NCT03421977, NCT04042025) have been initiated, enrolling participants who were treated with onasemnogene abeparvovec-xioi for SMA in the START study and previous (parent) studies for continuous monitoring of safety as well as monitoring of continued efficacy and durability of response to treatment. Despite a general favorable safety profile, some concerns have been raised, since at least three children with SMA developed thrombotic microangiopathy (TMA), after being treated with onasemnogene abeparvovec (Chand et al., 2021). All three children developed TMA approximately 1 week after treatment, and they had all contributory factors, including concurrent infections and recent vaccine exposure, which could be putatively contributing to development of TMA. Moreover, coagulation abnormalities have been reported more frequently in children with SMA (Wijngaarde et al., 2020), which could mean that they are more susceptible and precautions should be taken. They recovered after receiving plasmapheresis, high dose corticosteroids, and/or transfusions. TMA has also been reported with other gene therapies using AAV vectors including treatment of Duchenne muscular dystrophy (Chand et al., 2021). Since thrombocytopenia is a key feature of TMA, it is recommend that platelet counts are monitored after starting treatment with onasemnogene abeparvovec. Fairly recently, an indirect comparison has been attempted, which suggests that onasemnogene abeparvovec may have an efficacy advantage relative to nusinersen for overall survival, independence from permanent assisted ventilation, motor function, and motor milestones, when comparing clinical trials (NCT02122952 vs. NCT02193074) using frequentist and Bayesian approaches (Dabbous et al., 2019).

### Multiple System Atrophy (MSA)

MSA is a rare neurodegenerative disorder characterized by Parkinsonism, cerebellar ataxia, and autonomic failure, impacting on striatonigral, olivopontocerebellar, and autonomic systems, with an approximate prevalence around 2 in 100,000 (Chrysostome et al., 2004). The key pathological hallmark is the presence of glial cytoplasmic inclusions with insoluble proteinaceous filaments in the oligodendrocytes, and therefore MSA is regarded as an α-synucleinopathy along with Parkinson's disease and dementia with Lewy bodies. The etiology of MSA is largely unknown, although some emerging evidence suggests the involvement of mutations in the genes *SNCA, COQ2, MAPT, GBA1, LRRK2*, and *C9orf72* (Katzeff et al., 2019). Currently, there is no treatment targeting MSA, therapeutic management is based on symptomatic treatment. MSA patients may benefit from l-dopa for the symptomatic treatment of Parkinsonism symptoms, whereas physiotherapy remains the best therapeutic option for the ataxia (Perez-Lloret et al., 2015).

So far, no gene therapies targeting the potential pathological gene variants have been accelerated into clinical testing. However, consistent with promising pre-clinical data in Parkinson's disease models after vector-mediated overexpression of glial-derived neurotrophic factor (GDNF, Axelsen and Woldbye, 2018), Brain Neurotherapy Bio is preparing for a randomized, double-blinded, placebo-controlled phase 1 trial with symptomatic AAV2-GDNF gene therapy delivered bilaterally to the putamen of adult MSA patients (NCT04680065).

### Amyotrophic Lateral Sclerosis (ALS)

ALS consists of a group of rare neurological diseases that mainly involve neurons responsible for controlling voluntary muscle movement. The disease is progressive and worldwide affects about 5.4 in 100,000 (Chiò et al., 2013). ALS is characterized by progressive degeneration of motor neurons in the brain and spinal cord, causing individuals to gradually lose their strength and ability to speak, eat, move, and even breathe. Most people with ALS die from respiratory failure, usually within 3–5 years after symptoms first appear. About 5–10% of all ALS cases are familial due to mutations in more than a dozen genes. About 34% of all familial cases in Europeans are caused by defects in the *C9orf72* gene (most likely GoTF mutations; Mejzini et al., 2019). Another 15–30% of familial cases result from GoTF mutations in the gene encoding copper-zinc superoxide dismutase 1 (SOD1; Mejzini et al., 2019). Currently, there is no cure for ALS, and the two drugs approved by the FDA for treatment of ALS, riluzole, an anti-excitotoxic glutamate antagonist, and edaravone, a free radical/reactive oxygen species scavenger presumed to mitigate oxidative injury, are not effective at reversing disease progression, although the former has modest benefits on survival, and the latter may halt ALS progression during early stages (Bensimon et al., 1994; Jaiswal, 2019).

Different AAV vector-mediated and ASO strategies aiming at silencing *SOD1* or *C9orf72* as a therapeutic approach in familial ALS are currently being developed and tested in pre-clinical studies (Cappella et al., 2019; Amado and Davidson, 2021). Novartis Gene Therapies (formerly AveXis) has developed a self-complementary AAV9 vector expressing a short hairpin (sh) RNA to silence SOD1, and this vector has shown promising results after IV or intra-cisterna magna delivery in SOD1 mutant mice at different ages (Foust et al., 2013; Iannitti et al., 2018). Subsequently, the AAV9-sh-SOD1 vector was further tested successfully via subpial delivery in mice, pigs, and non-human primates (Bravo-Hernandez et al., 2020). Similarly, an AAVrh10 vector encoding artificial miRNA has been developed and delivered in a single intra-CSF injection in monkeys to silence the activity of the mutated SOD1, which significantly lowered SOD1 expression in spinal cord motor neurons, and the treatment was overall well-tolerated (Borel et al., 2016, 2018). Likewise, an AAVrh10-antisense-SOD1 vector was tested *in vivo* in adult SOD1 mutant mice through combined IV and ICV delivery (Biferi et al., 2017). The principle of targeting SOD1 expression with AAV-mediated down-regulation of malign SOD1 variants gained further support from a recently completed compassionate-use study in two SOD1-ALS male patients aged 22 and 56 years old (Mueller et al., 2020). After a single intrathecal injection of an AAVrh10 vector (4.2 × 10^14^ vector genomes) encoding miRNA targeting SOD1, the 22-year-old patient showed reduced post-mortem levels of SOD1 in spinal cord tissue and a transient improvement in right leg strength without change in vital capacity, whereas the 56-year-old patient who received immunosuppressive treatment had stable composite measure ALS functional scores and a stable vital capacity during 12 months. Safety assessment included vital signs, clinical laboratory assessments, CSF SOD1 activity and protein levels, electrocardiogram, physical and neurological examination, and any adverse events. This study suggests that intrathecal AAV-mediated miRNA gene therapy could be developed for treatment of SOD1-linked ALS, but potentially requires the concomitant use of immunosuppresants, and additional studies with larger numbers of patients are needed. Finally, focusing on *C9orf72* silencing, pre-clinical testing of an AAV5-miRNA-C9ORF72 vector sponsored by UniCure showed marked reduction of *C9orf72* transcripts in ALS mouse models (Martier et al., 2019).

### Huntington's Disease (HD)

This is an autosomal dominant disorder resulting from GoTF mutations in the form of CAG trinucleotide repeats in the *HTT* gene on chromosome 4p16.3 that encodes the widely expressed cytoplasmic protein, huntingtin (HTT, Jimenez-Sanchez et al., 2017). This leads to abnormal expansion of the polyglutamine sequence in HTT resulting in HTT aggregation that causes neurodegeneration, choreatic movements, as well as cognitive and behavioral disturbances. The prevalence of HD varies globally but overall affects around 2.7 in 100,000, and it commonly affects patients between the ages of 30–50 years, being most often fatal 10–15 years after diagnosis (Pringsheim et al., 2012). There is no cure for the disease, and current treatment is merely aimed at improving the quality of life and decreasing complications. With the treatment strategy to reduce the toxic effects of HTT, several clinical trials are ongoing with the use of ASOs for down-regulating HTT (Rodrigues and Wild, 2020), but recently disappointing results were reported from trials by Roche and Wave Therapeutics (Kingwell, 2021). To explore the potential of gene therapy, uniQure Biopharma, in 2019, has initiated a phase 1/2 randomized, double-blind, sham-controlled study to assess safety, tolerability and efficacy after multiple ascending doses administered into the striatum of AAV5-miHTT encoding miRNA aimed at reducing levels of HTT (AMT-130) in adult patients with early manifest HD (NCT04120493; Rodrigues and Wild, 2020).

## Neurodevelopmental Disorders

### Rett Syndrome (RTT)

RTT is a progressive neurodevelopmental disorder with multisystem comorbidities that occurs almost exclusively in girls, affecting an estimated 10–11.8 in 100,000 females (Ip et al., 2018; Kyle et al., 2018; Fu et al., 2020). Boys typically die shortly after birth. In its classic form of RTT, girls have 6 to 18 months of apparently normal development before developing often highly debilitating problems with learning, language, coordination, autism symptomatology, and epilepsy. Several variant forms of RTT have been described which can be milder or more severe than the classic form. Up to 95% of cases of RTT are caused by LoF mutations in the X-linked gene *methyl-CpG-binding protein 2* (*MECP2*), which is a ubiquitously expressed transcriptional regulator critical for normal brain function, including the maintenance of synaptic connections (Ip et al., 2018). It remains unclear how these changes lead to the specific features of RTT. Several conditions with signs and symptoms overlapping those of RTT have been found to result from mutations in other genes. These conditions, including forkhead box protein G1 (FOXG1) syndrome and cyclin-dependent kinase-like 5 (CDKL5) deficiency disorder, were previously thought to be variant forms of RTT but are now usually considered to be separate disorders.

There is no known cure for RTT, and treatment is directed at improving symptoms. Anticonvulsants may be used to help with seizures. Many of those with the condition live into middle age. Using MECP2-knockout mice, encouraging pre-clinical results with increased survival and body weight have been seen after intracisternal delivery of AAV vectors encoding MECP2 (Gadalla et al., 2017; Sinnett et al., 2017; Sandweiss et al., 2020). An alternative approach is the use of CRISPR/Cas9 genome editing that has been shown to be efficient at correcting FOXG1 variants in human RTT patient-derived fibroblasts and induced pluripotent stem-derived neurons using AAV9 vectors (Croci et al., 2020). Human clinical trials remain to be initiated.

### Genetic Epilepsy Syndromes

Epilepsy is one of the most common neurological diseases characterized by an enduring pre-disposition to generate seizures. Despite having an overall lifetime prevalence of approximately 800 in 100,000 (Beghi, 2020), it comprises a large heterogeneous group of syndromes of which some of them fulfill the definition of a rare disease in this review (i.e., <40–60 in 100,000), e.g., Dravet syndrome (severe myoclonic epilepsy in infancy; 2.5 in 100,000), Lennox-Gastaut syndrome (15 in 100,000), West syndrome (infantile spasms; 8 in 100,000), and Angelman syndrome (5–8 in 100,000) (Buiting et al., 2016; Döring et al., 2016). The majority of these genetic epilepsies are diagnosed within the first months to years of life with developmental deterioration or seizures as the first symptomatic signs. Since these syndromes are mostly drug-resistant, gene therapy could become an alternative treatment avenue (Turner et al., 2021).

The vast majority of Dravet syndrome cases are caused by a LoF mutation in one allele of the *SCN1A* gene, resulting in voltage-gated sodium channels with a non-functional NaV1.1 subunit primarily in inhibitory GABAergic neurons, leading to hyperexcitability and seizures associated with high risk of sudden infant death (Samanta, 2020). Pre-clinical data after single intrahippocampal injection of an AAV vector (ETX101) mediating increased production of functional copies of *SCN1A* in GABAergic interneurons in Dravet mouse model show decreased seizure frequency and severity as well as lower mortality (Steriade et al., 2020). Encoded Therapeutics is currently preparing for clinical trials using ETX101 for *SCN1A*-positive Dravet syndrome patients^1^. Genome editing is also being explored with CRISPR/Cas9-based gene therapy triggering SCN1A transcription in inhibitory neurons shown to ameliorate seizures in Dravet syndrome mice (Colasante et al., 2020; Yamagata et al., 2020). Encouraging reductions in seizures and mortality were also seen in a mouse model of Dravet syndrome after ICV administration of ASO that increases expression of SCN1A transcripts by reducing non-productive splicing (Han et al., 2020). A recently initiated clinical trial sponsored by Stoke Therapeutics will test this ASO (STK-001) in Dravet syndrome patients (NCT04442295). These studies also suggest that RNAi gene therapeutic vectors targeting non-productive splicing could become relevant.

Another largely monogenic rare genetic epilepsy syndrome is that of Angelman syndrome which is most often caused by LoF mutations in the maternal *UBE3A* gene encoding ubiquitin-protein ligase E3A (Turner et al., 2021). This *UBE3A* deficiency can be potentially treated by gene replacement therapy and, indeed, intrahippocampal AAV9-UBE3A injection caused some improvements in a mouse model of Angelman syndrome (Daily et al., 2011). However, there is some concern that a gene replacement strategy could be associated with side effects if UBE3A expression levels become too high since this might result in autism (Vatsa and Jana, 2018). An alternative strategy that has been explored is to activate the dormant UBE3A gene on the paternally inherited chromosome which is silenced by expression of paternal expression of UBE3A-ATS transcripts by the use of ASO treatment targeting UBE3A-ATS (Elgersma and Sonzogni, 2021). Currently, a clinical trial with intrathecal administration of an ASO (GTX-102) is ongoing using this approach^2^. If successful, it is possible that gene therapy with vectors similarly targeting UBE3A-ATS via RNAi constructs could also come into play.

Other rare genetic epilepsy syndromes, like Lennox-Gastaut and West syndromes, can be due to mutations in multiple different genes, and the molecular mechanisms of these mutations are poorly understood (Mastrangelo, 2017; Pavone et al., 2020). Consequently, gene therapeutic pre-clinical studies are so far limited. Since more than 100 causative genes have been identified in epilepsy syndromes (Helbig and Ellis, 2020), it can, however, be expected that gene therapy will attract increasing attention for treating genetic epilepsies in the near future. One example of a gene therapeutic approach was conducted with a RNAi vector (scAAV9-miDnm1a), targeting pathogenic *DNM1* gene variants in a mouse model of developmental and epileptic encephalopathy that prevented development of lethal tonic-clonic seizures (Aimiuwu et al., 2020). *DNM1* encodes a brain-specific GTPase, dynamin-1, which mediates presynaptic endocytosis, and the few individuals identified with pathogenic *DNM1* variants suffer from developmental and epileptic encephalopathy syndromes including Lennox-Gastaut syndrome and infantile spasms. Moreover, gene therapy trials with symptomatic vector construct approaches are also on the way targeting hyperexcitability in more frequent focal epilepsies with intracerebral injections of viral vectors mediating focal overexpression of engineered Kv1.1 potassium channel (NCT04601974; lentivirus; Snowball et al., 2019) or neuropeptide Y and its antiepileptic receptor Y2 (AAV1-NPY-IRES-Y2; CG01; Szczygiel et al., 2020; Cattaneo et al., 2021). It is likely that these gene therapy vectors could also be efficacious in genetic epilepsy syndromes, by targeting the general disease mechanisms underlying seizure development instead of single mutated genes *per se*.

## Lysosomal Storage Diseases

### Batten Disease

Batten disease is the common name for a broad class of rare, fatal, inherited neurodegenerative lysosomal storage diseases affecting the nervous system and often retina, also known as neuronal ceroid lipofuscinoses (CLNs; Johnson et al., 2019). Batten disease affects 2–4 in 100,000 live births (Santavuori, 1988) and has several forms (CLN1-CLN14; Specchio et al., 2021) that share some common features and symptoms but vary in severity and age when symptoms appear. Each form is caused by LoF mutations in different genes affecting lysosomal function. Most forms of Batten disease/CLNs begin during childhood where symptoms may include vision loss, seizures, loss of previously acquired skills, dementia, abnormal movements, and greatly shortened life expectancy.

Traditional medications are available as symptomatic treatment for symptoms such as seizures, anxiety, depression, parkinsonism, and spasticity. Alternative treatments being explored include enzyme replacement therapy (for CLN1 and CLN2 diseases), stem-cell therapy (for CLN1, CLN2, CLN8 diseases), and gene therapy (for CLN1-CLN3, CLN5-CLN8, CLN10, CLN11 diseases) in pre-clinical and clinical studies (Johnson et al., 2019; Liu et al., 2020; Specchio et al., 2021). Studies of potential treatments are lacking for CLN4, CLN9, and CLN12-CLN14 diseases. So far, enzyme replacement therapy with ICV-administered cerliponase alpha for CLN2 disease is the only one that has been approved for Batten disease (Markham, 2017).

#### CLN1 Disease

CLN1 is caused by a LoF mutation of the gene *palmitoyl-protein thioesterase-1* (*PPT1*) that encodes the enzyme of the same name, leading to accumulation of lipopigments within cells, resulting in neuroinflammation and -degeneration. ABO-202, a scAAV9 vector that carries the *PPT1* gene, is a promising candidate for intracerebral gene therapy for CLN1. Pre-clinical studies with ABO-202 as well as various other AAV vectors encoding PPT1 have shown increased survival and improvement of neurological function in CLN1 mouse models (Shyng et al., 2017; Liu et al., 2020). ABO-202 has been granted Orphan Drug and Rare Pediatric Disease designations by FDA and Orphan Medicinal Product Designation from EMA. A phase 1/2 clinical trial is anticipated to start in 2021^3^.

#### CLN2 Disease

CNL2 also known as “Late Infantile Neuronal Ceroid Lipofuscinosis (LINCL),” derives from a defect in the lysosomal gene *CLN2* encoding the enzyme tripeptidyl peptidase 1 (TPP1) (Kohlschütter and Schulz, 2016), resulting in the lysosomal accumulation of ceroid lipofuscin. Clinical trials completed and ongoing suggest promising effects of intraventricular enzyme replacement therapy (BMN190; Markham, 2017; Schulz et al., 2018), but gene therapy is also being explored (Liu et al., 2020). AAV vectors encoding human TPP1 induced cerebral enzyme expression and increased survival in a mouse model of CLN2 disease after intracerebral administration (Passini et al., 2006; Kohlschütter and Schulz, 2016), and a human clinical trial (NCT00151216) with infusion into 12 distinct cerebral locations in 10 children suggested slowing of disease progression (Worgall et al., 2008; Souweidane et al., 2010). Two subsequent phase 1/2 trials (NCT01161576, NCT01414985) have been completed using AAVrh10-CNL2 that appeared more promising in the mouse model (Sondhi et al., 2007) and showed long-term expression and acceptable safety profile in rats and non-human primates (Sondhi et al., 2012), but, so far, no results have been published.

#### CLN3 Disease

This type of Batten disease derives from mutations in the *CLN3* gene that encodes a lysosomal membrane protein called battenin, the function of which is poorly understood. Based on encouraging pre-clinical data from mice (Bosch et al., 2016), a phase 1/2 gene therapy open-label, single dose, dose-escalation clinical trial (NCT03770572) has been initiated in subjects with CLN3 disease to explore safety and efficacy of intrathecal delivery in the lumbar spinal cord region of a vector encoding the normal human *CLN3* gene (scAAV9.P546.CLN3; AT-GTX-502). Using a mouse model of CLN3 disease, intraocular gene therapy with AAV-mediated expression of CLN3 may also be efficacious for treating loss of vision by preventing decline in inner retinal function resulting from the death of rod bipolar cells (Holthaus et al., 2020).

#### CLN5 Disease

This is caused by mutations in a lysosomal protein encoded by the gene *CLN5* (Liu et al., 2020). Gene therapy using lentivector, AAV9, or scAAV9 encoding CLN5 has shown promising results in sheep with naturally occurring CLN5 disease (Mitchell et al., 2018). The vectors were injected intraventricularly and/or directly into the brain parenchyma, and the treated sheep retained neurological and cognitive functions. So far, no human clinical trials have been initiated.

#### CLN6 Disease

This condition results from mutations in an endoplasmic reticulum membrane protein encoded by the gene *CLN6* (Liu et al., 2020). ICV-administered scAAV9 encoding CLN6 drastically reduced pathology, improved memory, motor performance, and survival in CLN6 mutant mice (Cain et al., 2019). ICV delivery of scAAV9-CLN6 also slowed visual deterioration in CLN6 disease mice by preventing disease pathology in visual centers of the brain and retina (White et al., 2021). Intrathecal gene therapy with a similar vector was shown to be safe and efficiently induce expression in the brain and spinal cord in non-human primates (Cain et al., 2019). A phase 1/2 single dose study is ongoing to test intrathecal administration of scAA9-CLN6 (AT-GTX-501) in CLN6 disease children (NCT02725580) and a 15-year follow-up study will evaluate long-term safety and efficacy (NCT04273243).

#### CLN7 Disease

This condition can result from more than 35 different mutations in the *MFSD8* gene encoding CLN7, a lysosomal putative membrane transporter protein (Danyukova et al., 2018). Recently, a novel concept known as *N-of-1* treatment has emerged, which encompasses true personalized medicine and development of specific treatment for single patients (Mullard, 2020). This was showcased by the development within only 1 year of a new unique ASO treatment, milasen, for a young child, suffering from CLN7 due to a unique mutation that caused mis-splicing of MFSD8 (Kim et al., 2019). After dose escalation followed by maintenance dosing every 3 months, it was found that seizures were substantially decreased and several neurologic and neuropsychological subscores stabilized during 7 months after treatment (Kim et al., 2019). Mila's case has created new hope in rare and ultra-rare diseases where gene therapy can potentially be developed in cases where only one person in the world has a specific genetic mutation (Kim et al., 2019). As for CLN7 disease patients, most of them will probably not benefit from treatment strategies targeting RNA mis-splicing. Another line of clinical testing is currently recruiting for a phase 1 open-label, single-dose gene replacement therapy (AAV9-MFSD8) administered intrathecally into the lumbar spinal cord of pediatric patients with CLN7 Batten disease (NCT04737460).

#### CLN8 Disease

This is a condition caused by biallelic mutations in the gene *CLN8* which encodes an endoplasmic reticulum cargo receptor that regulates lysosome biogenesis (di Ronza et al., 2018). A single neonatal ICV injection of a scAAV-9 vector encoding human CLN8 has recently shown a successful degree of rescue in a mouse CLN8 model, as revealed by reduced histopathology, substantial behavioral improvement, and increased lifespan (Johnson et al., 2021). These data clearly encourage the testing of gene therapy for this disorder.

#### CLN10 Disease

CLN10 disease is caused by homozygous or compound heterozygous mutations of the *CTSD* gene encoding cathepsin D, an aspartic endoprotease ubiquitously distributed in lysosomes (Liu et al., 2020). Intracerebral administration of a mosaic AAV1/2 encoding CTSD into neonatal CTSD knockout mice increased lifespan and rescued brain pathology, and CLN10-associated visceral abnormalities as well as lifespan were further improved by peripheral vector treatment (Shevtsova et al., 2010; Liu et al., 2020).

#### CLN11 Disease

This adult onset disease is caused by homozygous or compound heterozygous mutations in the *GRN* gene that encodes the protein granulin implicated in lysosomal function (Liu et al., 2020). Heterozygous mutations are associated with frontotemporal dementia (Baker et al., 2006; Cruts et al., 2006). Pre-clinical studies with CLN11 gene therapy using GRN knockout mice have generated conflicting results with AAV1-GRN vector injected into prefrontal cortex showing improved pathology, also outside the injected region (Arrant et al., 2018), while ICV injection of AAV9-GRN using a different promoter was associated with severe hippocampal neurodegeneration (Amado et al., 2019). Further pre-clinical studies are needed to clarify the safety and efficacy of gene therapy with GRN overexpression for CLN11 disease.

### Krabbe Disease (KD)

KD is an autosomal recessive, often fatal lysosomal storage disease leading to pronounced neurodegeneration (Kwon et al., 2018). KD is also known as globoid cell leukodystrophy because of the characteristic multinucleated globoid cells found in a brain biopsy and the presence of white matter degeneration. The disease has an estimated prevalence around 1 in 100,000 (Foss et al., 2013). KD is caused by a LoF mutation at human chromosome 14, which codes for a lysosomal hydrolase known as galactosylceramide beta hydrolase (GALC), which is responsible for metabolizing galactolipids in both the central and peripheral nervous systems. KD disease is subdivided into sub-categories based on the age at presentation of symptoms. It is possible to screen newborns for KD, but current tests to identify which children are likely to develop the disease are inadequate (Kwon et al., 2018; Ehmann and Lantos, 2019). HSPC transplantation is the only available treatment for early infantile KD and should be performed before the onset of symptoms to be effective (Ehmann and Lantos, 2019).

Currently, promising pre-clinical data (Bradbury et al., 2018, 2021; Rafi et al., 2020) have enabled Forge Biologics to plan recruitment in 2021 of children below 12 months of age in a phase 1/2 clinical study, performing IV administration of an AAVrh10 vector expressing GALC (FBX-101) in newborns with early-infantile KD receiving HSPC transplantation (NCT04693598). A similar approach is applied by Passage Bio who intend to start recruitment in 2021 of 1–9 months children with early-infantile KD for treatment with intracisternal injections with the recombinant AAVhu68 vector encoding human GALC (PBKR03; NCT04771416). In both studies, the aim is to assess safety, tolerability and efficacy of escalating doses of AAVhu68-GALC gene therapy.

### Metachromatic Leukodystrophy (MLD)

MLD has an incidence of 1.4–1.8 in 100,000 live births (Rosenberg et al., 2016). Leukodystrophies are genetic disorders of the CNS leading to progressive neurologic deterioration; in the case of MLD, the disease arises from a deficiency of the lysosomal enzyme arylsulfatase A (ARSA) due to LoF mutations in the *ARSA* gene. This leads to a build-up of sulfatides, resulting in cerebral demyelination and loss of neurons. It affects both oligodendrocytes and Schwann cells, thus affecting neurons in both the central and peripheral nervous system. MLD is classified depending on age at onset of symptoms, the most common form, known as late infantile form, debuts at around 2 years of age (accounting for 50% of cases). Patients die within a few years after onset and display seizures, impaired swallowing, muscle wasting, paralysis, and dementia. Sulfatides accumulate in several other organs although the effect of this accumulation has not yet been observed. This could be attributed to the short lifespan of these patients, and accumulation of sulfatides could have effects that will be revealed if survival is increased (Rosenberg et al., 2016).

Gene therapy has been utilized *ex vivo* in combination with bone marrow transplants to re-implant genetically corrected HSPCs to patients (utilizing lentivectors and insertion of healthy genes into the cell genome) (Rosenberg et al., 2016). Three children with ARSA deficiency and mutations associated with early-onset MLD were included in a phase 1/2 trial, carried out in a partnership between Orchard Therapeutics and San Raffaele-Telethon Institute for Gene Therapy, and treated at the pre-symptomatic stage with autologous CD34-positive HSPCs transduced *ex vivo* with a lentivector carrying the *ARSA* gene (OTL-200; Biffi et al., 2013). This resulted in stable engraftment of transduced HSPCs at high levels and with reconstituted ARSA activity in the cerebrospinal fluid and arrested progression of neurodegenerative disease in all patients (Sessa et al., 2016; NCT01560182). Subsequently, preliminary data from 33 early-onset MLD patients with up to 7.5 years follow-up after treatment with OTL-200 suggested a favorable safety profile (no treatment-related mortality, no malignancies, no abnormal clonal expansion, and no evidence of replication-competent lentiviruses) and efficacy at modifying the disease course of early-onset MLD patients (hematological recovery, stable OTL-200 engraftment, ARSA activity restoration, and long-term stabilization of motor functions) as compared to a national history cohort (Calbi et al., 2020). Recently, OTL-200 was approved for treatment of MLD by EMA in 2020 under the tradename Libmeldy®, but OTL-200 is not yet approved by the FDA. In addition, two studies applying the same principle, but in later onset symptomatic MLD patients, have been initiated to evaluate short- and long-term safety and efficacy (NCT02559830, NCT04283227).

In addition, treatments with *in vivo* gene transfer are being explored. Based on promising pre-clinical results in rodents and non-human primates after intracerebral injection of an AAVrh10-ARSA vector that induced high expression of ARSA in neurons and oligodendrocytes (Piguet et al., 2012; Zerah et al., 2015), a phase 1/2 clinical study of ARSA gene transfer with 12 intracerebral injections to children with early onset forms of MLD was initiated in 2013 (Penati et al., 2017; NCT01801709). So far, results remain to be posted. Another phase 1/2 study sponsored by the Shenzhen Geno-Immune Medical Institute will test effects of ARSA gene therapy on MLD patients using a safety- and efficiency-improved self-inactivating lentivector (TYF-ARSA) after intracerebral injections (NCT03725670). Safety will be evaluated with regard to vital signs, physical examination, treatment-emergent adverse events, biochemical analysis, and magnetic resonance imaging (MRI) up to 3 years post-treatment.

### Mucopolysaccharidoses (MPS)

MPS are a defined group of different lysosomal storage disorders (MPS I-IX) caused by a deficiency in lysosomal enzymes catalyzing degradation of glycosaminoglycans (GAGs) that affects 4 in 100,000 (Poswar et al., 2019). GAGs consist of long chains of sugar carbohydrates aiding the buildup of bone, cartilage, tendons, corneas, skin, and connective tissue. Lysosomal enzyme deficiencies of MPS lead to aberrant development with neurocognitive and musculoskeletal pathological abnormalities. Each MPS is clinically heterogeneous, with milder to more severe cases within each type (Terlato and Cox, 2003). Diagnosis is determined by measuring urinary GAGs, enzyme activity in blood samples, and by identification of specific gene variants related to each MPS enzyme (Kubaski et al., 2020). Current treatment consists of enzyme replacement therapy (e.g., for MPS I and II; Concolino et al., 2018) and allogeneic HSPC transplantation (Poswar et al., 2019). However, despite reduced morbidity, these do not prevent persisting neurocognitive and musculoskeletal deficits (Fraldi et al., 2018). It is, therefore, not surprising that gene therapeutic approaches are under exploration. Here we will mainly describe the gene therapy efforts in the MPS types I, II, and III which show consistent CNS involvement. In other MPS types, e.g., VI, although clinical testing has been performed, CNS is not affected and, consequently, outside the scope of this review (for review see Fraldi et al., 2018).

#### MPS I

This type of MPS is divided into three subtypes (Hurler, Hurler-Scheie, and Scheie syndromes) based on severity of symptoms. All three are caused by a defective gene causing alpha-L-iduronidase (IDUA) enzyme deficiency and tissue accumulation of the GAGs heparan and dermatan sulfate (Hampe et al., 2021). Several encouraging studies have been performed using animal models of MPS type 1 with intrathecally or IV administered IDUA gene replacement approach using serotype 9 or rh10 AAV vectors, including in rodents, dogs, cats, and non-human primates (Watson et al., 2006; Hinderer et al., 2014, 2015; Belur et al., 2020). In line with these promising pre-clinical data, a first-in-human gene therapy study sponsored by Regenxbio has been initiated using an AAV9-IDUA vector (RGX-111) designed to restore IDUA enzyme activity in the brain (NCT03580083). This is an open-label, dose-escalation phase 1/2 study evaluating safety, tolerability, efficacy, and pharmacodynamics after intracisternal delivery of RGX-111 to MPS I patients during the 24 weeks study period. An ongoing *ex vivo* phase 1/2 clinical trial (NCT03488394) explores safety and efficacy of IV-injected autologous HSPCs genetically modified to express IDUA using a LV in patients with the Hurler variant. A long-term follow-up study will look for adverse neurologic and other events for up to 30 years after (NCT00695279). Genome editing has also been explored as a treatment approach. Both ZFN and CRISPR-Cas9 platform studies where AAV8 vectors were injected IV to genome edit liver cells have shown encouraging results in rodents (Ou et al., 2020; Poletto et al., 2020). The latter platform may be more efficacious (Ou et al., 2020), but, so far, only ZFN genome editing has entered clinical testing using an IV-injected AAV6 vector that inserts a corrective copy of the IDUA transgene into the genome of patients' hepatocytes (NCT02702115; Harmatz et al., 2019). This is expected to provide permanent, liver-specific expression of IDUA. A 10-year long-term safety follow-up study is also ongoing (NCT04628871). Although CNS symptoms were clearly improved in a mouse model of MPS I (Ou et al., 2020), it is disputed to what extent enzymes will pass into the CNS (Poletto et al., 2020). Nonetheless, it is suggested that constant high blood levels of IDUA in the blood may cause sufficient amounts to reach the brain (Ou et al., 2020).

#### MPS II

This type of MPS, also known as Hunter syndrome, results from a recessive X-linked LoF mutation in the gene encoding the lysosomal enzyme iduronate-2-sulfatase (IDS; Sestito et al., 2018). Pre-clinical models of MPS II have shown success with regard to improvement of neurological symptoms using gene replacement *ex vivo* after transplantation of HSPCs modified to synthesize IDS via a lentivector or *in vivo* using intracerebral administration of an AAV-IDS vector (Gleitz et al., 2018; Sestito et al., 2018). Two phase 1/2 open-label multicenter *in vivo* gene replacement trials (AAV9-IDS; RGX-121) are currently recruiting patients. One is a dose-escalation study (NCT03566043), and the other is a single dose study (NCT04571970). A follow-up study will evaluate long-term safety and efficacy of RGX-121 over 5 years (NCT04597385). Consistent with encouraging results with ZFN-mediated *in vivo* genome editing in mouse model of MPS II (Laoharawee et al., 2018), the first human genome editing trial (CHAMPIONS) in the form of a phase 1/2, multicenter, open-label, ascending dose trial is currently investigating efficacy of genome editing with the use of an AAV6 vector delivering ZFN (SB-913) that corrects the IDS gene in hepatocytes of MPS II patients, aiming to provide permanent, liver-specific expression of IDS (NCT03041324; Muenzer et al., 2019). An additional long-term safety study will follow the patients for 10 years (NCT04628871). As argued above for MPS I genome editing targeting liver cells, it remains to be seen to what extent CNS symptomatology will be improved by the treatment (Poletto et al., 2020).

#### MPS III

This type of MPS is also known as Sanfilippo syndrome and exists in five different forms (A-E) that are all recessive lysosomal storage diseases primarily affecting the brain (Pearse and Iacovino, 2020). MPS type IIIA causes Sanfilippo syndrome A and is the most common and severe type of MPS III with lowest survival rate (Pearse and Iacovino, 2020). The disease is caused by enzyme deficiency of N-sulfoglucosamine sulfohydrolase (SGSH; also known as heparan-N-sulfatase) due to LoF mutation in the *SGSH* gene, leading to the lysosomal accumulation of the GAG heparan sulfate (Winner et al., 2016). Promising results from pre-clinical studies with AAV-mediated gene transfer in animal models of MPS IIIA (Winner et al., 2016) led to initiation of phase 1/2 clinical trials in four children with MPS IIIA sponsored by Lysogene (Tardieu et al., 2014; NCT01474343, NCT02053064). The catalytic site of SGSH is activated by a sulfatase-modifying factor (SUMF1). Consequently, the vector that was injected intracerebrally in these trials encoded both SGSH and SUMF1 (AAVrh10-SGSH-IRES-SUMF1; LYS-SAF301). At 1-year follow-up moderate improvements were observed in three patients (Tardieu et al., 2014). The treatment was generally well-tolerated. An enhanced vector (AAVrh10-SGSH; LYS-SAF302) only encoding SGSH, the enzyme deficient in MPS IIIA, that induces 3-fold higher enzyme expression than LYS-SAF301 (Laufer et al., 2019) is currently being tested in an open-label single arm, phase 2/3 study after intracerebral delivery (NCT03612869; AAVance trial). A 5-year-old girl from this trial recently died several months after receiving the vector injection, and consequently the FDA has so far put the trial on hold while it is being analyzed whether the death is related to the gene therapy^4^,^5^. Two other trials using IV administration of a vector carrying the human *SGSH* gene (scAAV9-hSGSH; ABO-102) sponsored by Abeona Therapeutics are also currently recruiting (NCT02716246, NCT04088734). Long-term safety and efficacy will be monitored in a 5-year follow-up study (NCT04360265). Preliminary data suggest that ABO-102 is well-tolerated (Marcó et al., 2019).

Pre-clinical safety and efficacy of *ex vivo* transduction of CD34-positive HSPCs with a LV containing *SGSH* (LV-SGSH) has been demonstrated (Ellison et al., 2019). This has led to initiation of a phase 1/2 study using *ex vivo* gene therapy with LV-SGSH transduced CD34-positive HSPCs administered to MPS III A patients (NCT04201405). The study is sponsored in collaboration between the University of Manchester and Orchard Therapeutics (Kinsella et al., 2020).

MPS type IIIB causes Sanfilippo syndrome B due to deficient enzyme alpha-N-acetylglucosaminidase (NAGLU) activity (Pearse and Iacovino, 2020). A phase 1/2 clinical trial sponsored by UniQure Biopharma has tested gene replacement therapy using intracerebral administration of AAV5-hNAGLU in four MPS IIIB patients (NCT03300453). The treatment appears to be safe and well-tolerated with sustained NAGLU production in the CSF 30 months after injection (Tardieu et al., 2017). Abeona Therapeutics has sponsored a 2-year open-label, dose-escalation phase 1/2 trial using IV administration of an AAV9 vector encoding the human NAGLU gene (AAV9-hNAGLU; ABO-101; NCT03315182). No results are available yet.

MPS type IIID causes Sanfilippo syndrome D due to deficiency in N-acetylglucosamine 6-sulfatase (GNS; Pearse and Iacovino, 2020). GNS-deficient mice show lysosomal storage CNS pathology, locomotor deficits, and shortened lifespan similar to humans with MPS IIID, and intracisternal administration of a vector encoding GNS (AAV9-GNS) reversed these deficits (Roca et al., 2017). These encouraging results await clinical testing.

## Neurometabolic Disorders

## Canavan Disease (CD)

CD is a rare leukodystrophy resulting in neurodegeneration that occurs after a LoF mutation in the gene encoding aspartoacylase (ASPA). ASPA deacetylates N-acetylaspartate (NAA), and dysfunction results in accumulation of NAA in the nervous system (and in urine). The result of accumulation of NAA is dysmyelination, vacuolation of white matter and intramyelinic edema leading to hydrocephalus (Ahmed and Gao, 2013). The overall incidence is unknown. It occurs most frequently in individuals of Ashkenazi Jewish descent where it affects, at up to 16 in 100,000 (Zayed, 2015). ASPA mRNA is mainly found in oligodendrocytes while it is not present in neurons (Kirmani et al., 2002). NAA is produced in neurons and transported to the extracellular space where it is taken up by glial cells. NAA constitutes more than 0.1% of the healthy brain by weight, yet the function of NAA remains largely unknown, and the mechanism of CD pathology is unclear (Leone et al., 2012; Gessler and Gao, 2016; Gessler et al., 2017). Three subclasses exist based on onset of symptoms and severity of progression: Congenital, infantile and juvenile. CD is fatal in its congenital form where children die within days or weeks after birth (Ahmed and Gao, 2013, Gessler and Gao, 2016). Most patients suffer from the infantile form with symptoms including hypotonia, macrocephaly, blindness, and halting motor function development starting within the first postnatal months (Gessler and Gao, 2016).

The first pre-clinical gene therapy studies on CD utilized a lipid-entrapped, polycation-condensed delivery system in combination with an AAV-based plasmid encoding ASPA that was administered by intracerebral and intraventricular injections to healthy rodents and primates (Gessler and Gao, 2016). Subsequently, a proof-of-concept study with the same injection constructs was performed on two children with CD (Leone et al., 2000). Although the effect of this type of gene therapy was well-tolerated, and some biochemical and radiological parameters improved, no clinically relevant disease rescue was observed (Gessler and Gao, 2016). A few years later a follow up study conducted a phase 1 trial in a larger group of CD patients with an improved system for delivering the enzyme (AAV2-ASPA; Janson et al., 2002; Leone et al., 2012). A follow-up study found that AAV2-ASPA gene therapy slowed progression of brain atrophy, reduced seizures, and stabilized overall clinical status (Leone et al., 2012). No severe adverse events related to the administration of AAV2-ASPA into six intracerebral infusion sites were reported after a minimum of 5-years follow-up (Leone et al., 2012).

Subsequent pre-clinical studies with ASPA gene replacement therapy have shown phenotype rescue after systemic injection of AAV9 or other AAV serotypes (i.e., rh8, rh10) that are able to cross the BBB or after intracerebral injection of AAV-Cy5 with a promoter that specifically targets oligodendrocytes in CD mice (Gessler and Gao, 2016; von Jonquieres et al., 2018). Additional studies introducing human *ASPA* gene replacement into the astrocytes in mice has provided support for utilizing the astrocytes as a metabolic sink for clearing NAA (Gessler et al., 2017). Again, successful treatment was age-dependent, with mice receiving treatment shortly after birth showing greater improvement in motor functions and survival (Gessler and Gao, 2016). NAA ASO knockdown of expression of neuronal NAA synthesizing enzyme N-acetyltransferase 8-like in a mouse CD model also showed some effect for 2 months after administration on NAA levels (Hull et al., 2020). Using a novel capsid variant, AAV/Olig001, with oligotropism allowing the vector to mediate ASPA expression more specifically in oligodendrocytes (Francis et al., 2021), a phase 1/2 open label clinical trial sponsored by CureRareDisease LLC has recently been initiated with administration of a single ICV dose of AAV/Olig001-ASPA (NCT04833907). The trial will enroll 24 CD children aged 3–60 months.

## Niemann-Pick Disease

Niemann-Pick disease occurs in 5 in 100,000 live births in Europe (Gessler and Gao, 2016). All subtypes result from acid sphingomyelinase (ASM) deficiency causing increases in in metabolic intermediates including sphingomyelin and cholesterol (Salegio et al., 2012). Different subtypes have been described: Niemann-Pick disease type A presents with cognitive decline, loss of motor function, and hepatosplenomegaly. Rapid neurodegeneration leads to death within 3 years after birth (Samaranch et al., 2019). Niemann-Pick disease type B displays milder progression and symptoms show before adulthood and neurological symptoms are less common. Niemann-Pick disease type C affects trafficking of endocytosed cholesterol, and symptoms start before adulthood and include ataxia, cognitive dysfunction, and loss of language. Patients only reach 10–25 years of age (Gessler and Gao, 2016). Type A and type B Niemann-Pick disease have a residual ASM enzyme activity of 1–2% and 5–10%, respectively, suggesting that even marginal increases in ASM activity could lead to a therapeutic beneficial effect (Salegio et al., 2012).

Enzyme replacement therapy has been suggested as a viable treatment for the peripheral symptoms of patients suffering from type A and B. Clinical trials are underway (Samaranch et al., 2019). As these do not reach the CNS, gene replacement by injection into the lateral ventricles has been suggested. Studies performed in knockout mice lacking the *ASM* gene showed some phenotypic improvements, which was not reproduced in non-human primates where poor diffusion from the ventricles into the brain parenchyma limited the spread of the transgene. Injections into the brain parenchyma of AAV vectors encoding the *ASM* gene were more effective, though only at injection sites limited by poor spread of the transgene (Samaranch et al., 2019). A pre-clinical study with intracerebral delivery of AAV2-ASM in non-human primates resulted in only low spread of expression and toxicity in the form of immunogenicity as well as abnormal gait and posture and paresis in animals treated with high doses (Salegio et al., 2012). The immunogenicity and inflammatory response was suggested to arise from ASM-induced upregulation of cytokine CCL5 that is associated with gliosis and inflammation (Salegio et al., 2012). It is not clear to what extent CCL5 upregulation can limit the usefulness of *ASM* gene replacement therapy. Delivery has been also attempted via CSF through the cerebellomedullary cistern, resulting in transgene expression and better spread to deeper brain structures (Samaranch et al., 2019). Despite the initially promising opportunity of gene replacement, pre-clinical studies have revealed challenges related to ASM-induced calcium imbalance, aberrant intracellular signaling, inflammation and cell death (Lloyd-Evans et al., 2008), which should be carefully assessed and may be eliminated by dose de-escalation. No clinical trials utilizing gene therapy in humans have yet been initiated.

## X-linked Adrenoleukodystrophy (ALD)

ALD, also known as Lorenzo's oil disease, is an X-linked disease with an estimated incidence of 6.8 in 100,000 (Kemp et al., 2001). ALD is caused by a defective *ABCD1* gene that encodes a peroxisomal ATP-binding cassette transporter for transporting very long-chain saturated fatty acids (VLCFA) into the peroxisome for beta-oxidation. *ABCD1* dysfunction leads to a pathologic build-up of fatty acids damaging the myelin sheaths of the neurons in the brain, leading to cognitive and motor impairments (Turk et al., 2020). The disease primarily affects boys, though half of heterozygous females show some symptoms later in life. If left untreated, it will ultimately lead to a vegetative state and life expectancies of no longer than 10 years from time of diagnosis (Turk et al., 2020).

The *ABCD1* gene defect can be screened for in childhood genetic testing providing a short opening to start up treatment and to prevent the progressing and irreversible degenerative effects. More than 2,700 different *ABCD1* mutations have been identified, indicating a large degree of non-recurrent variations and *de novo* mutations and a low degree of phenotypic to genotypic correlation (Kemp et al., 2012; Turk et al., 2020). Current standard treatment for childhood cerebral ALD is allogeneic HSPC transplantation. However, this intervention is associated with high morbidity and long-term complications related to the concomitant chronic immunosuppression and graft-vs.-host response. Moreover, adrenal dysfunction is not corrected following the HSPC transplant for the cerebral disease.

One attempt to improve the clinical therapeutic options and decrease mortality is the execution of *ex vivo ABCD1* gene replacement in CD34-positive HSPCs in 17 ALD boys with early stage brain disease using a lentivector (Lenti-D) (NCT01896102; NCT03852498; Cartier et al., 2009; Eichler et al., 2017). The patient's own HSPCs were transduced *ex vivo*, inserting a correct version of the *ABCD1* gene into the patient's stem cells. Subsequently, the patients received chemotherapy to eradicate the host HSPCs and make room for the genetically altered cells, which were re-infused IV. As first indication, interim findings reported that 15 of the boys survived and remained free of major functional disabilities at the 2-years follow-up, with no treatment-associated death or graft-vs.-host disease reported. One boy died of rapid neurologic deterioration, and one withdrew from the study due to rapid disease progression and was instead submitted to allogeneic stem cell transplantation. A third clinical trial is recruiting advanced stage ALD patients to test the effects of *ABCD1*-corrected CD34-positive HSPCs (NCT02559830). A multi-center, long-term safety and efficacy follow-up study will test ALD patients treated with Lenti-D vector modified HSPCs for an additional 13 years (NCT02698579).

Pre-clinical *in vivo* gene therapy has shown promising results in a mouse model of ALD where intrathecal delivery of AAV9-ABCD1 in mice improved VLCFA metabolism and behavioral parameters (Gong et al., 2015). However, so far, only lentivector-mediated gene replacement approach administered intracerebrally to ALD patients is being tested in a Shenzhen Geno-Immune Medical Institute-sponsored phase 1/2 clinical trial using a self-inactivating LV (TYF-ABCD1; NCT03727555).

## Phenylketonuria (PKU)

PKU is a monogenic autosomal recessive disease caused by different LoF mutations in the *phenylalanine hydroxylase* (*PAH*) gene located on chromosome 12 with a prevalence between 6.7 and 10 in 100,000 newborns (Woo et al., 1983; Gessler and Gao, 2016). PAH catalyzes the hydroxylation of phenylalanine to tyrosine and is predominantly expressed in the liver but is also found in kidney, pancreas, and brain (Lichter-Konecki et al., 1999). The underlying disease mechanism for PKU is not fully understood, however, if left untreated, it leads to severe intellectual disability, developmental impairment, seizures, and psychosocial problems (White et al., 2010). Today's screening programs of newborn children and early nutritional intervention can reduce cognitive impairment. However, gene therapy has been explored at the pre-clinical level since gene replacement normalizing liver PAH activity and even boosting CNS PAH expression are predicted to potentially improve cognition and quality-of-life for PKU patients. Thus, both *ex vivo* and *in vivo* PAH gene replacement therapy in murine models of PKU (Gessler and Gao, 2016). For instance, *in vivo PAH* gene replacement by portal or tail vein injection in mice using an AAV8-PAH vector was associated with long-term reduction of phenylalanine levels with no elevation of markers of liver damage, inflammation, or humoral immune response against vector-mediated PAH expression (Ding et al., 2006; Harding et al., 2006).

Recently, translation into the clinic has begun with three trials currently recruiting. The first is a phase 1/2, open-label, randomized, dose-escalation study sponsored by Homology Medicines to evaluate the safety and efficacy for 1 year after a single IV-injection of an AAVHSC15 vector containing a functional copy of the human *PAH* gene (HMI-102; NCT03952156). This AAV serotype administered IV has been observed to cross the BBB and transduce neurons in the brain and spinal cord in non-human primates (Ellsworth et al., 2019) and could, consequently, further boost PAH CNS expression in addition to peripheral expression. A second clinical trial is a 5-year follow-up safety and efficacy study to the first trial (NCT04348708). The third trial, sponsored by BioMarin Pharmaceutical, is a phase 1/2 open-label, dose-escalation study which tests an AAV5 carrying a functional *PAH* gene (BMN 307; NCT04480567).

## Gangliosidoses

Gangliosidoses (GM1 and GM2) are neurodegenerative lysosomal storage disorders resulting from autosomal recessive mutations causing the accumulation of lipid gangliosides (Breiden and Sandhoff, 2019).

### GM1 Gangliosidosis

GM1 gangliosidosis results from LoF mutations in the *GLB1* gene, leading to deficiency in beta-galactosidase 1 (GLB1) hydrolase that results in GM1 ganglioside accumulation primarily in nervous tissue in the CNS. Incidence is estimated to be 0.5–1 in 100,000 (Tonin et al., 2019). Age of onset and progression of GM1 gangliosidosis differ, and the disease is divided into infantile (Type I), late-infantile/juvenile (Types IIa and IIb), and adult (Type III). The early forms constitute the more serious forms with multiple severe hallmark symptoms, including the typical CNS manifestations and severe cognitive and physical disabilities. The disease is uniformly fatal with no effective therapy and standard of care is limited to symptomatic medical management. Two clinical trials aiming at gene transfer with AAV vectors carrying a functional copy of the *GLB1* gene are currently ongoing. In 2019, a non-randomized, phase 1/2 clinical trial started aiming at evaluating the safety and efficacy of a single dose AAV9-GLB1 (AXO-AAV-GM-1) after IV infusion to subjects, aged 2–12 years old, with Type II GM1 gangliosidosis (NCT03952637). The study is conducted in a collaboration between the National Human Genome Research Institute and Axovant Sciences and is expected to be completed in 2023. Not long after in April 2020, Lysogene started another phase 1/2 clinical trial aiming at evaluating safety and efficacy of different doses of AAVrh10-GLB1 (LYS-GM101) infused IV to subjects with infantile Type I GM1 gangliosidosis (NCT04273269). This study is expected to be completed in 2025. Passage Bio is sponsoring a third phase 1/2, single-arm, dose escalation, multicenter study currently recruiting that will test an AAVhu68-GLB1 vector (PBGM01) delivered into the cisterna magna in infantile GM1 patients with Types I and IIa gangliosidoses (NCT04713475).

### GM2 Gangliosidoses

GM2 gangliosidoses include the Tay-Sachs disease, the Sandhoff disease, and the GM2 AB, which result from LoF mutations in the genes *HEXA, HEXB*, and *GM2A*, respectively (Dastsooz et al., 2018). These variants of GM2 gangliosidoses are clinically indistinguishable, but are all associated with beta-hexosaminidase deficiency (Leal et al., 2020). All three variants are usually fatal by early childhood. Tay-Sachs disease, which is the more common of the GM2 gangliosidoses (0.5 in 100,000; Meikle et al., 1999), debuts around 6 months of age and results in death by the age of 4 years. Sandhoff disease (0.25 in 100,000; Meikle et al., 1999) results from LoF mutations in the *HEXB* gene on chromosome 5, critical for the lysosomal enzymes beta-N-acetylhexosaminidase A and B. The GM2 AB variant is caused by gene mutations causing cofactor GM2 activator deficiency leading to lack of the normal beta-hexosaminidase A. With no authorized treatments available the current standard of care for GM2 gangliosidosis is limited to supportive care aimed at providing adequate nutrition and hydration. So far, no clinical gene therapy trials have been conducted in GM2 gangliosidoses. However, several pre-clinical approaches with intracerebral co-administration of AAV1, scAAV9.47, and AAVrh8 vectors have been used in Sandhoff disease mice and cats and in non-human primates to transfer the genes for the beta-hexosaminidase α and β subunits, which resulted in increased lifespan, reduced GM2 ganglioside levels, and improved motor functions (Cachon-Gonzalez et al., 2018; Leal et al., 2020). Recently, IV administered gene transfer of both the α and β subunits was performed using a bicistronic ssAAV9-HexBP2A-HexA vector, again leading to increased lifespan, reduced GM2 ganglioside brain levels, and improvement in motor performance in Sandhoff disease mice (Woodley et al., 2019). Furthermore, intracerebral administration of AAVrh8 vectors encoding the α and/or β subunits showed therapeutic effect in a Tay-Sachs disease sheep model, with slowing of disease progression and reversal of ganglioside accumulation (Gray-Edwards et al., 2018). In contrast, neurotoxic effects were observed in normal macaques after bilateral intra-thalamic infusion of a combination of two AAVr8 vectors encoding α and β subunits at three tested doses, suggesting that species differences exist with regards to effects of gene therapeutic regulations of *HEXA/HEXB* (Golebiowski et al., 2017). Nonetheless, two phase 1/2 clinical trials are currently recruiting GM2 patients for intrathecal treatment with an AAV9 carrying *HEXA* and *-B* genes (TSHA-101; NCT04798235) or bilateral intrathalamic and dual intracisterna magna/intrathecal administration of a mixture of AAVrh8-HEXA and AAVrh8-HEXB vectors (NCT04669535).

## Discussion

Great progress in gene therapy has been made over the last decade. From the approvals of Luxturna® (voretigene neparvovec), the first gene therapy against inherited eye diseases, Zolgensma® (onasemnogene abeparvovec) as treatment for SMA, and Libmeldy® for MLD, we stand on the brink of gene therapy to deliver on its promise to potentially cure or modulate severe diseases. The increasing number of development projects and progress highlighted in this review offers reasons for optimism toward novel gene therapy treatments being approved for rare genetic diseases of the brain and spinal cord in a foreseeable future. Fully aligned with these expectations the FDA has proclaimed that they expect to be approving 10–20 cell and gene therapies a year from 2025^6^, and it is likely that some of these will fall within rare genetic diseases of the brain and spinal cord. However, for this to happen it is essential to obtain an even better understanding of the biology, pharmacodynamics, and safety of the applied systems. This became increasingly evident during the 1990s with the occurrence of firstly a fatal case of acute immunogenicity induced by a human adenovirus (type 5) vector in an ornithine transcarbamylase trial (Raper et al., 2003), and secondly genotoxicity induced by treatment with a gamma-retro virus vector in SCID-X1 (Hacein-Bey-Abina et al., 2003). These events were not expected but have had far reaching effects not only on the affected patients but also on the trajectory of gene therapy research and clinical trials. This has underlined the requirements and importance of both short-term and long-term monitoring of efficacy and safety aspects (Wilson, 2009; Somanathan et al., 2020). Recently we were once again reminded of the intrinsic uncertainties in drug development, e.g., with the Audentes Therapeutics gene therapy for treatment of X-linked myotubular myopathy being cleared to resume by the FDA after it was temporarily stopped for carefully reviewing the cases of unexpected deaths^7^ (Nature Biotechnology, 2020), suggesting that studies involving high systemic doses of AAV vectors should be carefully monitored for similar toxicities (Hinderer et al., 2018). However, even intracerebral AAV administration may also cause potential risks as seen in Lysogene's phase 2/3 trial for MPS IIIA (AAVance) where an unexpected death has presently put this trial on hold, as described above. Since not all adverse events are discovered shortly after the treatment, and some could occur only many years later, long-term follow up studies are required to gain the full understanding of the gene therapy safety profiles. Thus, a long-term study over up to 10 years in dogs treated with AAV vectors systemically for hemophilia A showed that the AAV vectors inserted its own genome into genes of the dogs associated with cell growth that could potentially lead to malignancy (Nguyen et al., 2021). Still the progress made has shown that the current wave of gene therapies being developed appears safer and more promising than its predecessors (Bulaklak and Gersbach, 2020).

Most types of gene therapy up till now use the *in vivo* approach of administering vectors that transfer DNA sequences into cells of the nervous system. Nonetheless, *ex vivo* modified re-implanted genetically modified stem cells have shown promising results as evidenced by the EMA approval of Libmeldy® for MLD and completed or ongoing clinical trials in ALD and MLD. So far, genome editing holds an unredeemed potential in treating rare genetic diseases of the brain and spinal cord providing improved symptomatic alleviation or in some cases even a curative potential. Most progress has been seen with MPS I and MPS II, where *ex vivo* and *in vivo* genome editing strategies are promising and have even reached clinical testing (Table 2). Here, we have until now seen the ZFNs being utilized rather than CRISPR/Cas9 even though the latter can be more easily designed and implemented, which could be due to the concerns associated with the off-target effects seen with CRISPR/Cas9 (Merkle et al., 2015). In addition to potential viral vector-specific risks described above, Cas9-based *in vivo* genome editing also raises concerns as to potential immunogenic responses to a bacterial protein (Wang et al., 2015, 2020). Nonetheless, considering that rapid progress is being made with development and testing of new genome editing platforms (Poletto et al., 2020), it is likely that genome editing in the future will become relevant as a treatment strategy for several genetic CNS disorders (Lubroth et al., 2021). This would, however, require better understanding and solutions toward safety concerns related to treatment-induced immunogenicity (Shim et al., 2017) and expanded control of on- and off-target modifications (Mills et al., 2003).

Expanding the scope to include more broadly gene-affecting treatments, we could expect to see more development programs and approvals within gene therapy and ASOs. First line of successes appears to fall within gene replacement in diseases caused by LoF mutations as seen with Zolgensma® (onasemnogene abeparvovec) in SMA1, where we have also appreciated the viable ASO strategies affecting RNA-splicing to boost expression of healthy protein as seen with Spinraza® (nusinersen). ASOs in general have the advantage that they exert a transient effect which provides security in case of unforeseen safety issues, and the development paradigm of N-of-1 treatment has even shown that it can be advanced to clinical testing rapidly for compassionate usage, as evidenced with the success of milasen in CLN7 Batten disease developed within 1 year (Mullard, 2020). On the other hand, it does not hold a disease-modifying potential and renders the patient with life-long need for taking medication. Therefore, it is likely that gene therapy using viral DNA vectors encoding shRNA/siRNA/miRNA/antisense RNA will prove to be better suited for obtaining a more stable long-lasting effect without the need for repeated central injections as seen with ASOs. Already AAV vectors are being tested in clinical trials to reduce production of pathologic proteins resulting from GoTF mutations for ALS (AAVrh10-antisense-SOD1) and HD (AAV5-miHTT).

For the genetic diseases which are often inherited and with an early-onset of symptoms, it is crucial to initiate treatment as early as possible (often within the first year of life) in order not to lose valuable time and treatment opportunities (Gessler and Gao, 2016; Al-Zaidy et al., 2019a,b). For instance, in SMA, animal models strongly suggest a critical window for gene therapy to achieve effective rescue, and clinical trials on children with SMA1 show improved outcome when gene therapy is performed earlier in the disease course (Robbins et al., 2014; Govoni et al., 2018). The same was found in *post-hoc* analysis where the biggest motor improvements were obtained in infants with SMA1 treated at an early age, highlighting the value of newborn screening and early treatment (Lowes et al., 2019). Similarly, in CD, phenotype rescue is better in patients treated at a younger age (Gessler et al., 2017), and in MLD *ex vivo* gene therapy appears to predominantly work on pre-symptomatic or early-symptomatic patients (Penati et al., 2017).

To ensure development of treatments for rare disease, including gene therapies, biopharmaceutical companies are incentivized with the prospect of fast-track designations and longer market exclusivity. Nonetheless, current and future gene therapies are coming with very high prices, many-fold higher than for other treatment modalities, which is justified by the higher development costs and small market of rare diseases. This will most likely limit the number of patients being offered new treatments even though there is evidence of efficacy and safety. This also means that the biopharmaceutical companies will be forced to build strong cases on how they can provide value for money to receive market authorization and recommendations for public or insurance reimbursement differing from country to country. In the case of Zolgensma® for SMA, the total costs range from 4.2 to 6.6 million dollars (Malone et al., 2019), which could carry to high a cost to be offered to larger numbers of patients, especially in countries with tax-funded public health care systems and strong prioritization and decision-making authorities.

To evaluate the cost-benefit of Zolgensma® compared to Spinraza®, a model was created based on data from the clinical trials START (AVXS-101-CL-101) and ENDEAR (nusinersen). As the AVXS-101-CL-0101 trial had no deaths, life expectancy of patients was estimated based on the motor milestones achieved - Patients who achieved sitting were aligned with the survival of SMA type 2 patients who sit but never walk, and patients treated who achieved walking aligned with SMA type 3 patients, who have normal life expectancy (Malone et al., 2019). From the modeling and simulations based on parametric curves fitted to estimate the probability of patients dying during each model cycle, it was assumed that 50% of SMA patients treated with Zolgensma® will survive until the age of 35, while the corresponding simulation applied to the ASO Spinraza® showed only 50% survival to the age of 3 years (Malone et al., 2019). An increasingly common way to evaluate novel treatments is by applying the quality adjusted years (QALY) analysis, and in a comparative case between Zolgensma® and Spinraza® this yielded QALYs of 15.65 and 5.29, respectively (Malone et al., 2019).

These high costs should be evaluated in the prospects of increased QALY and lowered medicine and hospitalization expenses. Furthermore, the benefits and ethical considerations of allowing patients who would have died in early childhood to live well into adulthood are to be considered. The calculations above are based on simulations on current data since treatment has only been available for a few years and long-term data and results remain to be seen (Malone et al., 2019). Interestingly, we have seen new and atypical ways of drug development, as in the case of N-of-1 and the ASO treatment for Batten CLN7 disease, where it was possible to raise money on an individual basis. It will be important that developers and payers work together on new ways of orchestrating models of pricing and reimbursement to ensure that gene therapy treatments reach the patients in need.

In conclusion, we expect that gene therapy will become increasingly relevant for rare brain and spinal cord diseases in the coming future. Considering that the vast majority of medical treatments available for diseases of this review offer merely symptomatic alleviation without targeting the underlying pathological etiology, approval of more gene therapies by regulatory authorities could become game changers for patients affected by rare diseases. This also highlights the potential of a paradigm shift where we move from symptomatic alleviation to disease modification and even cure.

## Author Contributions

All authors contributed to the article and approved the submitted version.

## Conflict of Interest

DW is co-founder and consultant of CombiGene AB (Lund, Sweden) and CG is employed by UCB Nordic A/S (Copenhagen, Denmark). The remaining author declares that the research was conducted in the absence of any commercial or financial relationships that could be construed as a potential conflict of interest.

## Publisher's Note

All claims expressed in this article are solely those of the authors and do not necessarily represent those of their affiliated organizations, or those of the publisher, the editors and the reviewers. Any product that may be evaluated in this article, or claim that may be made by its manufacturer, is not guaranteed or endorsed by the publisher.

## Footnotes

^1^ *https://encoded.com/encoded-therapeutics-announces-135-million-series-d-financing-to-support-first-clinical-trials-in-scn1a-dravet-syndrome-and-advance-preclinical-pipeline-of-gene-therapies-for-debilitating-neurologic/*

^2^ https://ir.ultragenyx.com/news-releases/news-release-details/genetx-and-ultragenyx-announce-positive-interim-phase-12-data#

^3^ *https://www.globenewswire.com/news-release/2020/08/17/2079104/0/en/Abeona-Therapeutics-and-Taysha-Gene-Therapies-Enter-into-Licensing-and-Inventory-Purchase-Agreements-for-ABO-202-a-Clinical-Stage-Novel-One-time-Gene-Therapy-for-CLN1-Disease.html*

^4^ https://www.genengnews.com/news/lysogene-confirms-childs-death-in-phase-ii-iii-gene-therapy-trial/

^5^ https://www.firstwordpharma.com/node/1730262?tsid=17

^6^ *https://www.fda.gov/news-events/press-announcements/statement-fda-commissioner-scott-gottlieb-md-and-peter-marks-md-phd-director-center-biologics*

^7^ *https://www.audentestx.com/press_release/audentes-therapeutics-announces-fda-lifts-hold-on-aspiro-clinical-trial-of-at132-for-treatment-of-x-linked-myotubular-myopathy-xlmtm/*
